# [^18^F]FDG PET in conditions associated with hyperkinetic movement disorders and ataxia: a systematic review

**DOI:** 10.1007/s00259-023-06110-w

**Published:** 2023-01-27

**Authors:** Elze R. Timmers, Marrit R. Klamer, Ramesh S. Marapin, Adriaan A. Lammertsma, Bauke M. de Jong, Rudi A. J. O. Dierckx, Marina A. J. Tijssen

**Affiliations:** 1 Department of Neurology, University Medical Center Groningen, University of Groningen, PO Box 30.001, 9700 RB Groningen, the Netherlands; 2 Expertise Center Movement Disorders Groningen, University Medical Center Groningen (UMCG), PO Box 30.001, 9700 RB Groningen, the Netherlands; 3 Department of Nuclear Medicine and Molecular Imaging, Medical Imaging Center, University Medical Center Groningen (UMCG), University of Groningen, PO Box 30.001, 9700 RB Groningen, the Netherlands

**Keywords:** [^18^F]FDG PET, Hyperkinetic movement disorders

## Abstract

**Purpose:**

To give a comprehensive literature overview of alterations in regional cerebral glucose metabolism, measured using [^18^F]FDG PET, in conditions associated with hyperkinetic movement disorders and ataxia. In addition, correlations between glucose metabolism and clinical variables as well as the effect of treatment on glucose metabolism are discussed.

**Methods:**

A systematic literature search was performed according to PRISMA guidelines. Studies concerning tremors, tics, dystonia, ataxia, chorea, myoclonus, functional movement disorders, or mixed movement disorders due to autoimmune or metabolic aetiologies were eligible for inclusion. A PubMed search was performed up to November 2021.

**Results:**

Of 1240 studies retrieved in the original search, 104 articles were included. Most articles concerned patients with chorea (*n* = 27), followed by ataxia (*n* = 25), dystonia (*n* = 20), tremor (*n* = 8), metabolic disease (*n* = 7), myoclonus (*n* = 6), tics (*n* = 6), and autoimmune disorders *(n* = 5). No papers on functional movement disorders were included. Altered glucose metabolism was detected in various brain regions in all movement disorders, with dystonia-related hypermetabolism of the lentiform nuclei and both hyper- and hypometabolism of the cerebellum; pronounced cerebellar hypometabolism in ataxia; and striatal hypometabolism in chorea (dominated by Huntington disease). Correlations between clinical characteristics and glucose metabolism were often described. [^18^F]FDG PET-showed normalization of metabolic alterations after treatment in tremors, ataxia, and chorea.

**Conclusion:**

In all conditions with hyperkinetic movement disorders, hypo- or hypermetabolism was found in multiple, partly overlapping brain regions, and clinical characteristics often correlated with glucose metabolism. For some movement disorders, [^18^F]FDG PET metabolic changes reflected the effect of treatment.

**Supplementary Information:**

The online version contains supplementary material available at 10.1007/s00259-023-06110-w.

## Introduction

Positron emission tomography (PET) with [^18^F]fluoro-deoxy-2-D-glucose ([^18^F]FDG) enables noninvasive measurements of regional glucose metabolism in the brain [1, 2]. Local glucose metabolism is strongly coupled with neuro-synaptic activity and, in some brain regions, even with synaptic density [3–5]. Regional changes in [^18^F]FDG uptake indicates altered regional brain function, which may be due to either local pathology or neuronal dysfunction in unaffected tissue caused by dysfunction of a remote interconnected brain region [6]. Such regional changes may be identified by measuring increases or decreases in local cerebral [^18^F]FDG uptake in patients, compared with control subjects [1]. Changes in commonly affected brain regions of a patient group can be visualized after uptake normalization steps, which may result in a disease-related pattern of regionally impaired neuronal function. Beyond such univariate analyses [7], spatial covariance analysis additionally considers relationships between regional changes, yielding a more direct identification of changes in network activity [8, 9]. However, it is important to take into account that changes in regional cerebral blood flow (rCBF) might influence the [^18^F]FDG uptake as well, especially in studies using standard uptake values (SUV) as an outcome measurement. On the other hand, both regional metabolism and rCBF are coupled with regional neuronal activity.

Previous reviews on [^18^F]FDG PET in movement disorders have focused primarily on movement disorders such as idiopathic Parkinson’s disease and Parkinsonian syndromes, characterized by clinical hypokinesia as one of the cardinal symptoms [10–12]. As changes in glucose metabolism also have been studied extensively in hyperkinetic movement disorders (HMD), a starting point of the present systematic review was to relate distinct hyperkinetic symptoms with regional changes in [^18^F]FDG uptake in the brain using a transnosological approach. Although the authors realize specific tracers may be interesting for specific pathologies within hyperkinetic disorders, the authors specifically wanted to focus only on the most commonly available and hence used PET tracer, [^18^F]FDG. Only a limited number of studies with other tracers have been performed, and focusing on [^18^F]FDG will minimize the heterogeneity of the results. Moreover, as indicated above, [^18^F]FDG allows assessment of a common parameter in all included studies, i.e., a spatial distribution of regionally changed neuronal function, which would not be the case for specific receptor tracers.

Patients with HMD suffer from an excess of involuntary movements [13]. The main characteristics of HMD are tremors, tics, dystonia, chorea, and myoclonus. We also included ataxia, although strictly it is not an excessive involuntary movement but rather a coordination problem [14]. In clinical practice, ataxia is usually taken together with the HMD. For that reason, we decided to include ataxia in our review. Many patients may have more than one movement disorder, and the same movement disorder can occur in a variety of neurologic disorders. The underlying pathophysiology of most movement disorders is not exactly known [13]. Given the heterogeneity of HMD, we followed a transnosological approach by selecting the main hyperkinetic symptoms often expressed in these HMD, and aimed to identify common regional brain alterations associated with these symptoms. In this way, patterns of symptom-specific regional brain alterations may contribute insight into disease-associated pathophysiology. To further gain insight into this association, the relationship between altered metabolism and clinical variables, including the severity of motor symptoms, will be discussed. Furthermore, [^18^F]FDG PET studies that evaluated the treatment of HMD will be reviewed. The latter studies are expected to provide insight into the relationship between the effect of a specific treatment and the brain areas involved.

## Methods

### Search strategy

A systematic literature search was performed according to PRISMA guidelines in order to identify all studies using [^18^F]FDG PET brain scans in HMD [15]. We included studies on tremors, tics, dystonia, ataxia, chorea, myoclonus, and functional movement disorders. In Table 1, a short symptom description is given [14, 16–19]. A combination of Medical Subject Headings (MeSH) terms and free text (supplementary data) was used to select all articles listed in PubMed until November 2021. Only original articles published in the English language with at least five patients were reviewed. Studies concerning additional HMD in patients with Parkinson’s disease or other parkinsonian syndromes were excluded, as studies on HMD result from structural brain lesions. Supplementary Fig. 1 shows a flow diagram of our inclusion process, independently performed by two authors (ET and MK).

**Table 1:** Description of movement disorders

Hyperkinetic movement	Brief description
Tremor	Involuntary, rhythmic, and oscillatory movements may involve one or several body parts
Tics	Repeated, individually recognizable, intermittent movements or movement fragments that are almost always briefly suppressible and are usually associated with awareness of an urge to perform the movement
Dystonia	A movement disorder is characterized by sustained or intermittent muscle contractions causing abnormal, often repetitive, movements, postures, or both
Ataxia^1^	Incoordination of balance, gait, extremity and eye movements, and dysarthria
Chorea	The ongoing random-appearing sequence of one or more discrete involuntary movements or movement fragments
Myoclonus	A sequence of repeated, often nonrhythmic, brief shock-like jerks due to sudden involuntary contraction or relaxation of one or more muscles
Functional movement disorder	Abnormalities of movement that are altered by distraction or nonphysiologic maneuvers and are clinically incompatible with movement disorders associated with neurologic disease

Description of the movement disorders discussed in this review [14, 16–19]. ^1^Ataxia is strictly not an excessive involuntary movement but rather a coordination problem; however, studies about patients with ataxia were included in this review as well

### Labeling of brain areas

Articles used a variety of brain atlases to label brain regions. In order to give a clear overview of the brain regions involved, we transformed the regions into a standardized template based on the automatic anatomical labeling (AAL) atlas (Supplementary Table 1) [20].

### Reporting of results

Results of [^18^F]FDG PET scans will be discussed per phenotype. Studies concerning patients with more than one movement disorder will be discussed in the section on the most prominent movement disorder. We additionally reported studies concerning patients with HMD due to an autoimmune- or metabolic disorder separately. In these diseases, a wide variety of movement disorders may be expressed in the affected patients. Although this prohibited a straightforward symptom classification, the FDG-PET findings do provide complementary information serving further understanding of the pathophysiological mechanisms involved. Results will be presented under three subheadings: (1) changes in regional metabolism compared with one or more control groups, (2) correlation between metabolism and clinical characteristics, and (3) effect of treatment on [^18^F]FDG PET.

## Results

### Search results

Of the 1240 studies retrieved in the original search, 104 articles were included in this review (Supplementary Table 1). Most articles concerned chorea, ataxia, dystonia, and tremors (Table 2). No papers on functional movement disorders were detected.

**Table 2:** Overview of number of articles per movement disorder and diagnosis

Movement disorder	Number of articles	Diagnosis (number of articles)
Tremor	8	Essential tremor (7)
		Orthostatic tremor (1)
Tics	6	Tourette syndrome (6)
Dystonia	20	Essential blepharospasm (5)
		DYT-TOR1A (5)
		DYT-THAP1 (3)
		Idiopathic cervical dystonia (3)
		Dopa-responsive dystonia (2)
		DYT-SGCE (1)
		Neurodegeneration with brain iron accumulation (1)
		Meige syndrome (1)
		Focal, segmental, and/or generalized dystonia (4)
		(Tardive dyskinesia in schizophrenic patients (1))
Ataxia	25	Spinocerebellar ataxia (13)
		Olivopontocerebellar atrophy (7)
		Multi system atrophy of the cerebellar type (4)
		Late cerebellar cortical atrophy (1)
		Ataxia-telangietactasia (1)
		Holmes type hereditary ataxia (1)
		Not further specified cerebellar ataxia (1)
		(Ataxia in patients with cryptogenic tonic–clonic seizures from infancy (1))
Chorea	27	Huntington’s disease (27)
		Chorea-acanthocytosis (1)
Myoclonus	6	Juvenile myoclonus epilepsy (3)
		Lafora disease (1)
		Myoclonus after a cardiac arrest (1)
		Myoclonus epilepsy with ragged red fibres (1)
Functional disorder	0	
Metabolic disease	7	Wilson’s disease (3)
		Niemann–Pick disease type C (1)
		Salla disease (1)
		Gluteric aciduria (1)
		Galactosemia (1)
Autoimmune disorder	5	LGI1-antibody encephalitis (2)
		Fisher’s syndrome (1)
		NMDA-ab encephalitis (1)
		Stiff person syndrome or cerebellar ataxia associated with anti-GAD65 antibodies (1)

Most studies compared a patient group with a group of healthy controls (*n* = 76) and/or with another patient group (*n* = 30). Some studies performed a correlation analysis between metabolism and clinical variables ((*n* = 46), most often motor severity (*n* = 35)), or evaluated the effect of treatment on glucose metabolism in the same patient group (*n* = 14).

### Tremor

Eight articles on patients with either essential tremor (ET) (*n* = 7) or orthostatic tremor (*n* = 1) were included in this review [21–28]. Sample sizes ranged from 5 to 42 patients.

### Changes in regional glucose metabolism

**Fig. 1:**
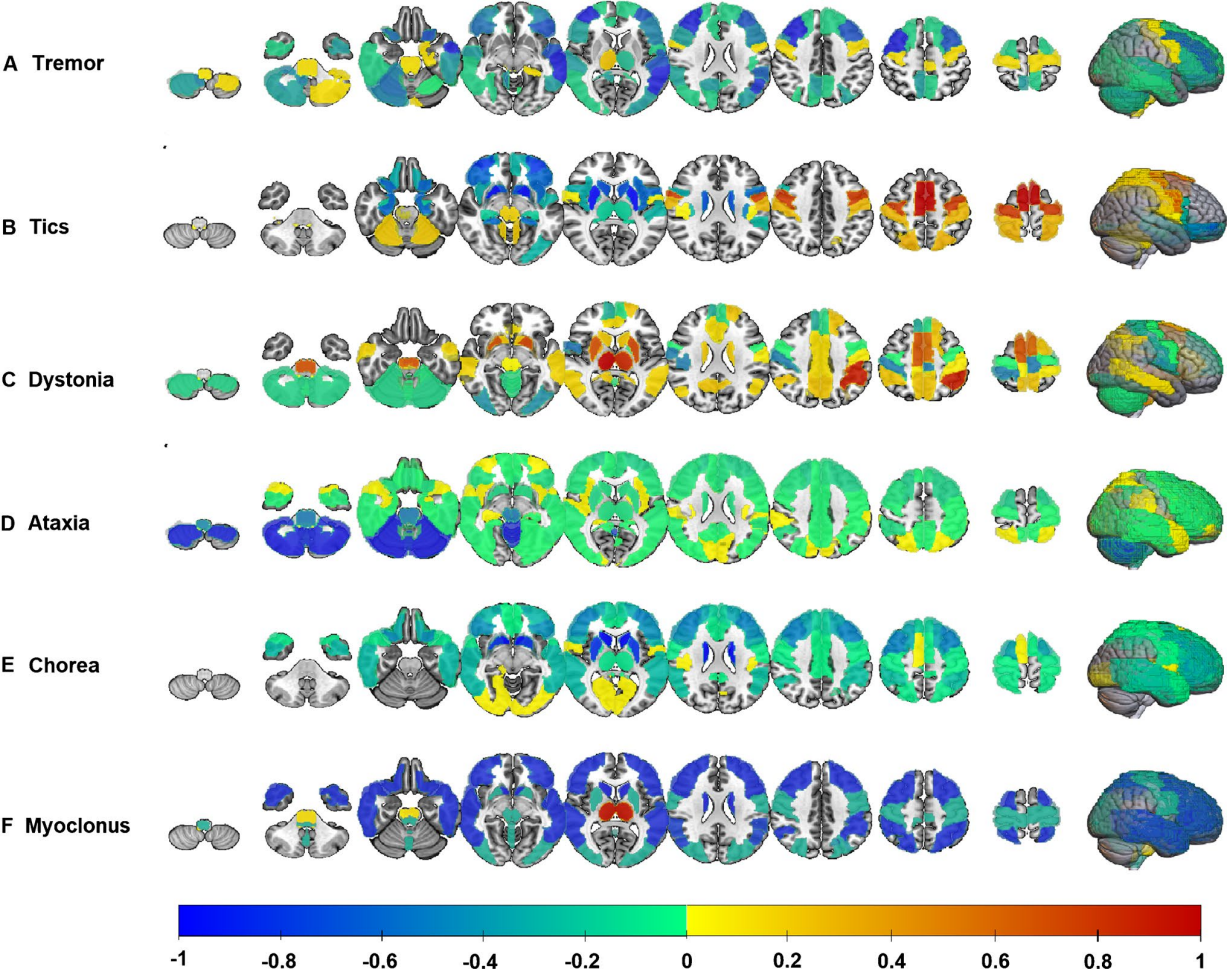
Regions that showed a significant difference between patients and healthy controls per hyperkinetic movement disorder. Negative values indicate that hypometabolism was found, and positive values indicate hypermetabolism. Data is normalized by the number of patients participating in each study, indicating that regions with a value of 1 or − 1 were found to be significantly different from healthy controls in the largest number of patients. Note that the choreatic pattern that is shown is only based on patients with HD. Due to the lack of group studies, no patients with acute chorea were included

Five articles comparing ET patients with healthy controls [21–25] reported hypometabolism in frontal- and temporal lobes (*n* = 3), precuneus (*n* = 2), and cerebellum (*n* = 2) (Fig. 1 and Supplementary Table 2). Schöberl investigated 10 patients with orthostatic tremor, lying versus standing, and compared them with controls, showing hypermetabolism in the brainstem, cerebellum, thalamus, frontal cortex, and parahippocampal gyrus [23].

### Correlation with clinical variables

Only two articles performed a correlation analysis between clinical variables and metabolism [27, 28]. In the first, following transcutaneous afferent patterned stimulation in five ET patients, no correlation was found between a change in motor severity and a change in metabolism (Table 3) [27]. The other study reported a negative correlation between body sway and metabolism in the mesiofrontal cortex in patients with orthostatic tremors when standing for 10 min after tracer injection [23].

**Table 3:** Correlation between regional metabolism and motor symptoms

Authors (year)	Movement disorder	Patients	Correlation with motor symptoms
Barath et al. [27]	Tremor	Patients with ET (*n* = 5) before and after treatment with TAPS	No correlation between the change in motor severity and change in metabolism
Eidelberg et al. [32]	Tics	Patients with Tourette syndrome (*n* = 10)	+ with subject scores of the pattern II No correlation with pattern I
Pourfar et al. [33]	Tics	Patients with Tourette syndrome (*n* = 12)	No correlation between symptom severity and Tourette pattern
Chase et al. [38]	Dystonia	Patients with idiopathic torsion dystonia (*n* = 6)	No correlation with motor severity
Eidelberg et al. [39]	Dystonia	Patients with idiopathic torsion dystonia (*n* = 11)	ROI analysis: no correlation with motor severity; SSM analysis: + with disease pattern subject score
Esmaeli-Gutstein et al. [51]	Dystonia	Patients with essential blepharospasm (*n* = 10) and Meige syndrome (*n* = 1)	No correlation with motor severity
Suzuki et al. [47]	Dystonia	Patients with drug induced blepharospasm (*n* = 21) and essential blepharospasm (*n* = 21)	No correlation with motor severity
Suzuki et al. [49]	Dystonia	Patients with essential blepharospasm (*n* = 39)	− with posterior and anterior striate cortex; + with the thalamus
Szyszko et al. [52]	Dystonia	Patients with DYT-TOR1A (*n* = 2), DYT-SGCE (*n* = 2), primary dystonia without genetic diagnosis (*n* = 12), and neurodegeneration with brain iron accumulation (*n* = 12)	− with motor severity and lingual gyrus + with dystonia duration and right and midline superior frontal gyrus
Aguiar et al. [58]	Ataxia	Patients with SCA36 (*n* = 20)	- with cerebellar hemispheres, vermis, and brainstem
Brockmann et al. [59]	Ataxia	Patients with SCA17 (*n* = 5)	+ with metabolic index
Gilman et al. [42]	Ataxia	Patients with OPCA (*n* = 30)	No correlation with age at the onset or duration of ataxia
Ishibashi et al. [75]	Ataxia	Patients with SCA (*n* = 12) subdivided into SCA6 (*n* = 3), SCA19/22 (*n* = 3), sporadic SCA (*n* = 6)	No significant correlation with motor severity
Korinthenberg et al. [74]	Ataxia	Patients with cryptogenic tonic–clonic seizures from infancy (*n* = 30) with ataxia (*n* = 15)	+ with the severity of ataxia and the frontal cortex; + with myoclonus and the temporal mesial area
Lee et al. [57]	Ataxia	Patients with MSA-C (*n* = 41)	− with motor severity and disease duration and orbitofrontal area, medial frontal, dorsal midbrain, and cerebellum
Manes et al. [76]	Ataxia	Patients with SCA38 (*n* = 10)	No correlation between (change in) motor severity and change in cerebellar metabolism
Meles et al. [63]	Ataxia	Patients with SCA3 (*n* = 17)	+ with SCA3-related pattern
Oh et al. [73]	Ataxia	Patients with MSA-C (*n* = 44), SCA2 (*n* = 9)	− with symptom duration in the left cortex (MSA-C) and right cortex (SCA2) of the cerebellum
Soong et al. [68]	Ataxia	Patients with SCA6 (*n* = 7)	No correlation with age at the onset or duration of ataxia
Volkow et al. [69]	Ataxia	Patients with ataxia-telangiectasia (*n* = 10)	− with the globus pallidus, vermis, thalamus, and precuneus
Wang et al. [70]	Ataxia	Patients with SCA2 (*n* = 8), SCA3 (*n* = 12), SCA6 (*n* = 7)	− with the frontal cortex
Ciarmiello et al. [83]	Chorea	Patients with advanced HD (*n* = 47)	No correlation with disease progression
Esmaeilzadeh et al. [101]	Chorea	Patients with HD (*n* = 8)	− after treatment with putamen, occipital-, temporal-, parietal-, and prefrontal cortex
Kuhl et al. [86]	Chorea	Patients with manifest HD (*n* = 13)	No correlation with disease duration or severity
Martínez-Horta et al. [103]	Chorea	Patients with early-mild stage HD who were bilingual (*n* = 30)	− with disease severity and left inferior orbitofrontal cortex
Sampedro et al. [106]	Chorea	Patients with early-stage HD (*n* = 18)	− with the left caudate
Squitieri et al. [100]	Chorea	Patients with HD treated with placebo (*n* = 12)	− with frontal, parietal and occipital cortex
Young et al. [97]	Chorea	Drug-free patients with early to midstage HD (*n* = 15)	− with chorea, dysdiadochokinesia, bradykinesia/rigidity, and striatum. + with dystonia and thalamus
Berkovic et al. [107]	Myoclonus	Patients with myoclonus epilepsy and ragged red fibers (*n* = 5)	No correlation with the severity of myoclonus
Hermann et al. [114]	Metabolic disease	Patients with Wilson’s disease (*n* = 37)	Patients with neurological symptoms had cluster I or III patterns, not correlated to severity motor symptoms
Kuwert et al. [115]	Metabolic disease	Patients with Wilson’s disease (*n* = 14)	− with severity extrapyramidal symptoms and caudate nucleus
Lau et al. [116]	Metabolic disease	Patients with Niemann–Pick disease type C (*n* = 14)	− with the frontal lobe
Schlaug et al. [117]	Metabolic disease	Patients with Wilson’s disease (*n* = 18)	− with the severity of neurological, striatal, and dystonia symptoms and the striatum + with the duration of neurological symptoms and striatum
Kim et al. [120]	Auto-immune	Patients with Fisher’s syndrome (*n* = 10)	− with days from symptom onset and cerebellum

Overview of articles who performed a correlation analysis with motor symptoms and metabolism. Results are given as a positive ( +) or negative ( −) correlation with motor severity (or otherwise specified) and metabolism in specified region. The following abbreviations are used: *ROI*, region of interest; *SSM(/PCA)L*, scaled subprofile model (principle component analysis); *GSOM*, growing self-organizing maps; *OPCA*, olivopontocerebellar atrophy; *SCA*, spinocerebellar ataxia; *MSA(–C)*, multi system atrophy (of the cerebellar type); *HD*, Huntington’s disease; *ET*, essential tremor; *TAPS*, transcutaneous afferent patterned stimulation

### Evaluation of treatment

Four studies evaluated the effect of treatment on [^18^F]FDG PET scans (Table 4) [24, 25, 27, 28] with the following results: (i) Comparing [^18^F]FDG PET scans before and 90 days after transcutaneous afferent patterned stimulation revealed increased metabolism in the ipsilateral cerebellar hemisphere and a decrease in the contralateral hemisphere [27]. (ii) Decreased metabolism was seen in the basal ganglia of eight ET patients who responded to propranolol treatment compared with nine nonresponders [24]. Apparently, different pathophysiological mechanisms play a role in the two groups. (iii) In line with this observation, a different pattern of hypometabolism, both before and after surgery, was found in 35 patients who responded to gamma knife of the ventral intermediate nucleus compared with seven nonresponders (Table 4) [25]. (iv) An increase in metabolism in the thalamus and cerebellum of five patients who developed gait ataxia after bilateral DBS of the (sub)thalamic area was shown during DBS ON, compared with scanning after 72 h of DBS OFF [28], while in five patients without gait ataxia, only a thalamic increase was observed. This indicates that the development of ataxia is related to DBS activation of connections with the cerebellum.

**Table 4:** Effect of treatment on regional metabolism

Authors (year)	Movement disorder	Outcome	Intensity normalization	Patients	Treatment	Time-point scans	Results
Barath et al. [27]	Tremor	SUV	Pons	Patients with ET (*n* = 5)	Transcutaneous afferent patterned stimulation	Baseline and 90 days	Ipsilateral cerebellar hemisphere↑; contralateral cerebellar hemisphere↓
Reich et al. [28]	Tremor	SUV	Global mean	Patients with ET (*n* = 10) treated with bilateral thalamic DBS of which some developed gait ataxia (*n* = 5)	Bilateral DBS of (sub)thalamic area	First scan with DBS-ON; second scan after 72 h with DBS-OFF	ET patients with ataxia (ON vs OFF): thalamus↑; cerebellum↑; ET control (ON vs OFF): thalamus↑
Song et al. [24]	Tremor	SUV	Not described	Male patients with essential tremor who responded to medical therapy (*n* = 8) and those who did not (*n* = 9)	Propranolol	6 months after treatment	Responders vs nonresponders: left basal ganglia↓
Verger et al. [25]	Tremor	Not clearly described	Global mean	Patients with right ET (*n* = 42) both responders (*n* = 35) and nonresponders (*n* = 7) to Gamma knife treatment	Gamma knife of left ventral intermediate nucleus	Baseline and after surgery	Before and after surgery: left thalamus↓, right cerebellum↓, left temporal gyri↓, frontal gyri↓; Responders vs nonresponders (before surgery): right retrosplenium↓, posterior cingulate cortex↓; Responders vs nonresponders (after surgery): right temporo-occipital area↓
Esmaeli-Gutstein et al. [51]	Dystonia	Not clearly described	Mean value of plane in which ROI was defined	Patients with essential blepharospasm (*n* = 10) and Meige syndrome (*n* = 1)	Botulinum toxin injections	Baseline and 1 or 2 weeks	No change in metabolism
Lalli et al. [45]	Dystonia	SUV	Global mean	Patients with cervical dystonia (*n* = 5)	Epidural premotor stimulation	Baseline and 12 months	Sensorimotor area↓
Manes et al. [76]	Ataxia	SUV	Global mean	Patients with SCA38 (*n* = 10)	Docosahexaenoic acid	Baseline and 40 weeks	Posterior cerebellar lobe↑
Manes et al. [78]	Ataxia	SUV	Global mean	Patients with SCA38 (*n* = 9)	Docosahexaenoic acid	Baseline and 104 weeks	Left exterior cerebellar lobe↑
Tsai et al. [77]	Ataxia	SUV	Not described	Patients with SCA3 (*n* = 6) and MSA-C (*n* = 1)	Mesenchymal stem cells	Baseline, 90 and 270 days	90 days: 2 patients with no changes, 3 patients overall glucose metabolism↓, 2 patients overall glucose metabolism↑;270 days: 6 patients overall glucose metabolism↑, 1 patient overall glucose metabolism ↓
Bachoud-Lévi et al [99]	Chorea	CMR_glu_	Global mean	Patients with HD (*n* = 5)	Fetal striatal grafting	Before right side graft (0 months), before left side graft (12 months), after 24 months	24 months vs. 0 months: caudate nucleus↑ and putamen↑ in 3 out of 5 patients
Esmaeilzadeh et al. [101]	Chorea	CMR_glu_ using an estimated input function	Not described	Patients with HD (*n* = 8)	Pridopidine	Baseline and 14 days	Precuneus↑, superior temporal gyrus↑, left middle frontal gyrus/premotor area↑, left medial dorsal nucleus of the thalamus↑
Kremer et al. [98]	Chorea	CMR_glu_	Global mean	Patients with HD (*n* = 26)	Lamotrigine (*n* = 14) and placebo (*n* = 12)	Baseline, 12, 24 and 30 months	Placebo vs. treated: Basal ganglia↓, frontal- and temporal cortex↓, thalamus↓
Paganini et al. [102]	Chorea	SUV	Cerebellum	Patients with HD (*n* = 10)	Fetal striatal grafting	Baseline, 2 and 4 years	2-year vs baseline: striatum↑; 4-year vs baseline: striatum↑
Squitieri et al. [100]	Chorea	SUV	Global mean	Patients with HD (*n* = 23)	Riluzole (*n* = 11) and placebo (*n* = 12)	Baseline and 24 months	Placebo vs. treated: change in all cortical areas↓

Overview of articles that investigated the effect of treatment on ^18^FDG PET scans. Results are shown as significant hypermetabolism (↑) or hypometabolism (↓) in the specified region. The following abbreviations are used: *SUV*, standardized uptake value; *CMR*_*glu*_, cerebral metabolic rate of glucose; *SCA*, spinocerebellar ataxia; *HD*, Huntington’s disease; *ET*, essential tremor; *DBS*, deep brain stimulation

### Key characteristics

[^18^F]FDG PET studies on tremors mainly involved patients with ET, while one study concerned orthostatic tremors. Most often, hypometabolism was reported in the cerebellum, frontal and temporal lobes, and precuneus. Although no studies found a correlation between motor severity and glucose metabolism, [^18^F]FDG PET uptake could predict the effects of treatment.

### Tics

All six [^18^F]FDG PET articles about tics included patients with Tourette syndrome. Three studies, all with different outcome measures, were from the same research group and most likely included, at least in part, the same group of patients [29–31]. The sample size ranged from 10 to 18.

### Changes in regional glucose metabolism

Four studies compared patients with Tourette syndrome with healthy controls [30, 32–34]. The most frequently reported findings were hypometabolism in the striatum (*n* = 4) and orbitofrontal cortex (*n* = 3), and hypermetabolism in the premotor cortex (*n* = 4) (Fig. 1). One study did not find differences between 10 patients and healthy controls using univariate analysis but did find two covariance patterns related to Tourette syndrome [32]. The first one distinguished patients with Tourette’s from healthy controls, but there was no relationship with disease severity. The subject scores of the second pattern were not significantly different between both groups, but in this case, a relationship with disease severity was observed (Supplementary Table 2). More recently, a study from the same group revealed a hypometabolic pattern comprising the orbitofrontal cortex and striatum, together with hypermetabolism in the premotor cortex and cerebellum, distinguishing patients from controls, but again, this pattern was not correlated with symptom severity [33].

A study on connectivity between different brain regions in both patients and healthy controls showed disruption of the connectivity of the ventral striatum and alterations in the limbic-motor interaction [29].

### Correlation with clinical variables

As mentioned above, a covariate pattern involving the caudate and lentiform nucleus, thalamus, and hippocampus was related to the severity of tics [32].

The severity of obsessive–compulsive symptoms was correlated with a network consisting of the cingulate, prefrontal and primary motor cortex, and precuneus [33]. Another study showed a positive correlation between complex cognitive and behavioral features and metabolism in the orbitofrontal cortex and putamen [31].

### Evaluation of treatment

No studies investigating the effect of treatment on [^18^F]FDG uptake in tics were identified.

### Key characteristics

All studies concerning tics in patients with Tourette syndrome found hypometabolism in the striatum and hypermetabolism in the premotor cortex. One disease-related pattern involved the caudate and lentiform nucleus, thalamus, and hippocampus, which was related to the motor symptoms. Frequently reported hypometabolism of the (orbito-)frontal cortex is most likely related to cognitive and behavioral features in Tourette’s syndrome.

### Dystonia

We included nineteen articles describing dystonia patients with a variety of diagnoses: idiopathic or drug-induced blepharospasm, hereditary dystonia (DYT-GCH1, DYT-TOR1A, DYT-THAP1, and DYT-SGCE), idiopathic cervical dystonia, and a heterogenous group with focal, segmental, and/or generalized dystonia patients [35–53]. One additional study reports on [^18^F]FDG PET scans in schizophrenic patients who developed tardive dyskinesia [54]. Sample sizes ranged from 5 to 50 patients.

### Changes in regional glucose metabolism

Fifteen articles compared dystonia patients with healthy controls [35–41, 43–49, 53]. The areas with altered glucose metabolism were quite heterogenous between groups. Articles describe hypermetabolism, and in a few studies hypo-metabolism, in the putamen (*n* = 7), globus pallidus (*n* = 6), pons (*n* = 4), and thalamus (*n* = 5), mainly hypometabolism in the cerebellum (*n* = 6) and hypermetabolism in the SMA (*n* = 4) (Fig. 1 and Supplementary Table 2).

The group of Eidelberg performed several studies using network analysis in order to find a dystonia-related pattern. Two studies, on dopa-responsive dystonia (*n* = 7) and idiopathic dystonia (*n* = 11), respectively, found a disease-related pattern involving the lentiform nucleus, brainstem, (pre)motor areas, and SMA in both dystonia groups. The dopa-responsive dystonia pattern additionally involved the vermis of the cerebellum [35, 39]. In seven non-manifesting DYT-TOR1A mutation carriers, a pattern of hypermetabolism in the cerebellum, basal ganglia, and SMA and hypometabolism in the midbrain was found [40]. In 10 manifesting DYT-TOR1A patients, subject scores of this pattern were higher than in healthy controls, indicating that this pattern was related to the DYT-TOR1A mutation [40]. However, applying this pattern to patients with essential blepharospasm also yielded significantly higher subject scores in patients compared with controls [44], which suggests that it is a more general dystonia-related pattern.

Results of studies comparing subtypes of dystonia or dystonia with ET patients can be found in Supplementary Table 3 [36, 37, 50].

### Correlation with clinical variables

In six articles, a correlation analysis between motor severity and cerebral glucose metabolism was performed. Three did not find any correlation (Table 3) [38, 47, 51]. A study assessing 11 idiopathic torsion dystonia patients did not find a correlation in univariate analysis but did find a positive correlation with the detected disease-related pattern and motor severity [39]. Two studies found a negative correlation between motor severity and metabolism in the visual cortex and lingual gyrus and a positive correlation with metabolism in the frontal cortex and thalamus [49, 52].

In DYT-TOR1A, seven non-manifesting and 10 manifesting patients had a common pattern of hypermetabolism in lentiform nuclei, cerebellum, and SMA, compared with normal controls, while a second pattern of hypermetabolism in the cerebellum, midbrain, and thalamus was seen in patients with lasting dystonia, compared with either non-manifesting patients, patients with dystonia in action only, or normal controls [40]. Two other studies reported regional hypermetabolism when comparing manifesting and non-manifesting patients with DYT-SGCE and DYT-TOR1A, respectively [36, 37]. Manifesting patients with an *SCGE* mutation had higher left cerebellum metabolism, and manifesting DYT-TOR1A patients had hypermetabolism in several brain areas.

### Evaluation of treatment

Two studies assessed the effects of botulinum toxin treatment in blepharospasm patients and epidural premotor stimulation in idiopathic cervical dystonia patients on regional [^18^F]FDG uptake (Table 4) [45, 51]. Scans before and after injection of botulinum toxin did not show any change in metabolism. Comparison before and 12 months after epidural premotor stimulation showed a significant reduction in hypermetabolism in the sensorimotor cortex, thus indicating a modulatory effect of this treatment [45].

### Key characteristics

All studies concerned chronic forms of dystonia. The lentiform nucleus, cerebellum, brainstem, thalamus, and SMA were affected most frequently. Several studies showed dystonia-related metabolic patterns, some related to the severity of motor symptoms. However, half of the studies could not find a correlation between motor symptom severity and metabolism in specific brain regions. In addition, treatment did not always have an effect on [^18^F]FDG uptake.

### Ataxia

Twenty-five articles concerning patients with ataxia were included [55–79]. Patients were diagnosed with spinocerebellar ataxia (SCA), olivopontocerebellar atrophy, multi-system atrophy of the cerebellar type (MSA-C), late cerebellar cortical atrophy, ataxia-telangiectasia, Holmes type hereditary ataxia, or unspecified cerebellar ataxia. The number of patients per study ranged from 5 to 46. Most studies did not perform a partial volume correction, except for the study of Oh et al. and Wang et al. [70, 73] However, some studies did not find atrophy or a correlation between atrophy and [^18^F]FDG uptake [61, 63, 67].

### Changes in regional glucose metabolism

The 19 articles that compared ataxia patients with healthy controls most frequently reported hypometabolism in the cerebellum (*n* = 19) and in the brainstem (*n* = 13) [55–73]. Relative hypometabolism was also shown in the frontal areas and the striatum (Fig. 1).

Nine studies made comparisons between patient groups or compared affected with non-affected patients or relatives (Supplementary Table 3) [55–57, 62, 67, 69, 70, 73, 79].

### Correlation with clinical variables

Four out of 12 studies assessing the relationship between motor severity and metabolism did not find any correlation [55, 68, 75, 76]. The other eight report an association between the severity of motor symptoms and metabolism in various brain areas, including but not limited to the cerebellum, frontal cortex, and brainstem (Table 3 and Supplementary Table 4) [57–59, 63, 69, 70, 73, 74].

### Evaluation of treatment

Two articles from the same research group investigated the short- and long-term effects of docosahexaenoic acid in patients with SCA38 (Table 2) [76, 78]. [^18^F]FDG PET after 40 weeks of treatment showed increased metabolism in the posterior cerebellar lobe, compared with pre-treatment metabolism, which persisted after 104 weeks. However, in the article, no arterial blood sampling was used to measure absolute glucose metabolism.

A study on the effect of mesenchymal stem cells measured using [^18^F]FDG PET in patients with SCA3 (*n* = 6) and one MSA-C patient described both decreases and increases in global glucose metabolism after 90 days [77]. After 270 days, six patients showed an increase in SUV, indicating some effect of the treatment. However, since the authors used SUV as an outcome measure, no robust statements can be made about variances in absolute glucose uptake.

### Key characteristics

Studies on several chronic types of ataxia were included. Hypometabolism in the cerebellum was a common feature. In addition, hypometabolism may occur in various regions, such as the brainstem, frontal cortex, and striatum. Motor symptoms were correlated with metabolism in these areas, and [^18^F]FDG PET scans showed potentially positive effects of treatment.

### Chorea

The 27 articles that reported on [^18^F]FDG PET in patients with Huntington’s disease (HD) had a number of symptomatic patients per study that ranged from 5 to 47 [80–106]. One study reported on a heterogeneous group with, next to HD patients, two patients with chorea-acanthocytosis, two patients with a HD phenotype but a without a positive family history, and two patients with hemichorea due to vascular lesions [95]. Although we excluded HMD due to structural lesions, we decided to include this study as the results are given to the whole group. Nine of the 27 articles performed a partial volume correction [82, 83, 85, 87, 100, 103–106].

### Changes in regional glucose metabolism

Eighteen articles compared patients with healthy controls. The most prominent finding was hypometabolism of the caudate nucleus and putamen (*n* = 18 and *n* = 15) [80–97]. Hypometabolism was also found in the frontal, temporal, and parietal regions, while relative hypermetabolism was observed in the occipital cortex and around the calcarine fissure (Fig. 1 and Supplementary Table 2).

### Correlation with clinical variables

Twelve studies performed correlation analysis with motor symptoms (*n* = 7) and/or other variables (Table 3 and Supplementary Table 4) [80–83, 86, 87, 97, 100, 101, 103, 105, 106]. Although hypometabolism in the striatum is generally thought to be related to motor symptoms in HD patients, only three studies reported a significant negative correlation between motor severity and metabolism in the caudate nucleus and/or putamen [97, 101, 106]. Three studies also found a correlation between motor severity and metabolism in the (pre-)frontal, temporal, parietal, and/or occipital cortices [100, 101, 103].

Some studies (*n* = 5) compared HD patients, in various stages of the disease, with premanifest HD patients or persons at risk for HD [81, 90, 96, 104, 106]. Again, hypometabolism of the caudate nucleus and putamen in the manifest HD patients was the most common finding (*n* = 5, Supplementary Table 3). A network analysis by the group of Eidelberg on [^18^F]FDG uptake in 18 premanifest HD patients and healthy controls revealed a disease-related pattern, characterized by hypometabolism of the striatum and medio-temporal areas and hypermetabolism in the occipital cortex [84]. This pattern did not only discriminate premanifest patients from controls, but subject scores of the pattern were significantly higher in manifest than in premanifest patients.

Scores of psychological and cognitive tests were most often correlated with metabolism in frontal and temporal areas, but also in the caudate nucleus and putamen (Supplementary Table 4) [80, 82, 101, 103, 105]. One study found hypometabolism in the orbitofrontal and inferior prefrontal cortex in four depressed HD patients compared with five HD patients that were not depressed [93].

### Evaluation of treatment

Three studies investigated the effect of medication on HD patients (Table 4). In all studies, [^18^F]FDG PET was performed before and after treatment. Compared with placebo, treatment with lamotrigine or riluzole resulted in less reduction in metabolism in the basal ganglia, frontal- and temporal cortex, thalamus, and/or all cortical areas. This indicates a protective effect on the neuronal functioning of these drugs [98, 100]. The final smaller study (*n* = 8) performed [^18^F]FDG PET before and 14 days after treatment with pridopidine, showing an increase in metabolism in the precuneus, temporal- and frontal cortex, and thalamus [101].

Two studies investigated the effect of fetal striatal grafting [99, 102]. Post-surgical scans in both studies showed an increase in glucose metabolism in the striatum compared with the presurgical scans, suggesting a good effect of grafting.

### Key characteristics

All studies included HD patients with chronic chorea. Hypometabolism in the striatum was the most common finding, which was correlated with the severity of motor symptoms. Hypometabolism in several cortical areas, including the frontal- and temporal cortex, was also frequently reported, and this was correlated with a decline in cognitive and behavioral functions, and also with motor symptoms. [^18^F]FDG PET was often used for measuring the effectiveness of treatment.

### Myoclonus

Six studies about myoclonus were included [107–112]. Five studies included patients with juvenile myoclonus epilepsy, myoclonus epilepsy with ragged red fibers, or Lafora disease (*n* = 19, 10, 9, 5, and 8) [107, 109–112]. Although these disorders do not solely encompass myoclonus, next to epilepsy, myoclonus is an important hallmark. Another small study (*n* = 7) with myoclonus after cardiac arrest was also included [108].

### Changes in regional glucose metabolism

All articles compared patients with healthy controls, and four found involvement of the thalamus [107–112]. The frontal and/or temporal cortex also showed a significant difference in metabolism in three articles (Fig. 1) [107, 108, 111, 112]. However, both hyper- and hypometabolism were reported (Supplementary Table 2).

One study did not find any difference between patients and healthy controls but found hypometabolism in the frontal lobe in patients with frontal lobe epilepsy compared with patients with juvenile myoclonus epilepsy (Supplementary Table 3) [110].

### Correlation with clinical variables

In patients with myoclonus epilepsy with ragged red fibers, no correlation was found between glucose metabolism and the severity of myoclonus [107].

For results of correlation analyses with EEG and executive functioning in patients with juvenile myoclonus epilepsy, see Supplementary Table 4 [109, 110].

### Evaluation of treatment

No studies investigated the effect of treatment on [^18^F]FDG uptake in myoclonus patients.

### Key characteristics

In all studies but one, myoclonus patients also suffered from epilepsy. The remaining study concerned (acute) myoclonus following cardiac arrest. Both hypo- and hypermetabolism of the thalamus were reported, with the two larger studies showing hypermetabolism. In addition, hypometabolism in several cortical areas was reported. No clear correlations between the severity of myoclonus and glucose metabolism were found.

### Metabolic disorders

Seven studies on patients with movement disorders resulting from a metabolic disorder were included [113–119]. Three studies concerned Wilson’s disease (*n* = 14, 18, and 37), in which patients had symptoms of dystonia, tremor, and ataxia [114, 115, 117]. Another study described patients with Niemann–Pick disease type C (*n* = 14) who suffered from ataxia (*n* = 13) and/or dystonia (*n* = 10) [116]. Ataxia was also the main symptom in patients with Salla disease (6 out of 9 patients) [118]. The last two studies described glutaric aciduria patients (*n* = 8), with dystonia, rigidity, and choreoathetosis and galactosemia patients (*n* = 5), with tremor, dystonia, and ataxia [113, 119]

### Changes in regional glucose metabolism

In six articles, patients with a metabolic disorder were compared with healthy controls [113–118], resulting in very heterogeneous areas with significantly changed metabolism (Supplementary Table 2). A network analysis of [^18^F]FDG uptake in 37 Wilson’s disease patients showed two patterns (both involving the cerebellum, midbrain, caudate nucleus, and thalamus) that discriminated patients with neurological symptoms from those without [114]. In another study, OD 18 Wilson’s disease patients showed reduced metabolism in the striatum of patients who were severely affected compared with mildly affected patients [117].

### Correlation with clinical variables

Two articles on Wilson’s disease found a negative correlation between the severity of motor symptoms and metabolism in the caudate nucleus or striatum [85, 117]. Furthermore, the duration of neurological symptoms was correlated with metabolism in the striatum as well. In patients with Niemann–pick type C, a negative correlation was found between motor severity and metabolism in the frontal lobe [116].

### Evaluation of treatment

Only one pilot study performed sequential PET scans in three patients with Wilson’s disease who were treated with D-penicillamine [117]. This study found an increase in [^18^F]FDG uptake in the striatum in two of these patients.

### Key characteristics

Significantly altered regional cerebral metabolism was found in patients with HMD due to metabolic disorders, but the pattern was quite heterogeneous, which is consistent with the heterogeneity of the underlying chronic disorders. Most studies concerned Wilson’s disease, of which one reported a network analysis showing two patterns that discriminated patients with neurologic symptoms from those without. Moreover, several studies showed a correlation between motor symptoms and metabolism in certain brain regions.

### Autoimmune disorders

Five articles included patients with HMD due to autoimmune disease [120–124]. The first article reported on 10 patients with Fisher’s syndrome, with all but one having ataxia [120]. Another article included patients with stiff person syndrome (*n* = 22) or cerebellar ataxia (*n* = 8) associated with anti-GAD65 antibodies [123]. Fifty-eight percent of patients in a study on cerebral metabolic changes in NMDA-antibody encephalitis had a movement disorder, either hyperkinetic (*n* = 6), hypokinetic (*n* = 3), or mixed (*n* = 17) [122]. Finally, two studies on patients with LGI1-antibody encephalitis reported facio-brachial dystonic seizures in 69 and 39% of patients, respectively [121, 124]. Only the sub-analysis on the 39% of patients with dystonia were used in this review [124].

### Changes in cerebral metabolism

Four articles performed a comparison with healthy controls [120, 122–124]. Results were, just as for the metabolic diseases, quite heterogeneous (Supplementary Table 2). Patients with Fisher’s syndrome (*n* = 10) and cerebellar ataxia associated with anti-GAD65 antibodies (*n* = 8) showed hypermetabolism in the cerebellum, which was also observed in 33 patients with NMDA-antibody encephalitis [120, 122, 123]. Hypermetabolism was also described in many cortical areas. In nine patients with facio-brachial seizures in LGI1-antibody encephalitis, only putamen hypermetabolism was found [124].

### Correlation with clinical variables

In 10 patients with Fisher’s syndrome, a negative correlation was found between the duration of symptoms and metabolism in the cerebellum [120]. The severity of disability was correlated with metabolism in part of the temporal cortex of eight patients with anti-GAD65 antibodies [123].

### Evaluation of treatment

No studies evaluated the effect of treatment on [^18^F]FDG uptake.

### Key characteristics

Similar to HMD due to metabolic disorders, the results of [^18^F]FDG PET studies in patients with autoimmune disorders were quite heterogeneous. However, the nature of these symptoms was more acute. In patients with ataxia, particularly hypermetabolism of the cerebellum was described. Altered metabolism in the basal ganglia and several cortical areas was also reported frequently, correlating, in some cases, with motor symptoms.

## Discussion

This review provides a comprehensive overview of [^18^F]FDG PET studies in patients with HMD. Brain regions with significant hypo- or hypermetabolism were described, and typical patterns are shown in Fig. 1. For each of the addressed symptoms, we found a common pattern of involved brain regions, despite the heterogeneity of the HMD etiologies. Furthermore, correlations with motor symptoms or other clinical variables and treatment effects were discussed.

Most [^18^F]FDG PET studies on tremors involved patients with ET, except for one study on orthostatic tremors. ET is believed to originate from the olivo-cerebellar circuits [125]. In line with this, most [^18^F]FDG studies found hypometabolism in the cerebellum, and only one study reported hypometabolism in the medulla oblongata in eight ET patients [22]. fMRI studies, similar to the [^18^F]FDG findings, found evidence that the cerebello-thalamic-cortical circuitry is also involved in the pathophysiology of ET [126]. In addition to this, H2 ^15^O PET studies showed involvement of the cerebellum and thalamus as well, however, these studies found an increased activity in these areas, which is seemingly in contrast to the low [^18^F]FDG uptake found in the cerebellum [127, 128]. This need; however, not be necessarily contradictory because one study concerned a H2 ^15^O PET activation study in which perfusion was assessed during evoked tremor compared to perfusion in a control condition of passive movement [127], thus assessing condition-related responses of a network. Evidence for metabolic alterations in the thalamus is limited and non-conclusive, with one study describing thalamic hypermetabolism in eight ET patients and one article with 42 ET patients reporting hypometabolism only in the left thalamus [22, 25]. Unfortunately, no [^18^F]FDG PET connectivity studies were performed, which are a better tool for assessing the involvement of circuitries.

All studies on tics concerned patients with Tourette syndrome. A previous transcranial magnetic stimulation study reported increased excitability of the cortical motor cortex, consistent with hypermetabolism of the premotor cortex described in [^18^F]FDG PET studies [129]. Several disease-related patterns were found, of which one, involving the caudate, lentiform nucleus, thalamus, and hippocampus, was also related to the tics [32]. In line with this, other functional studies showed that tics most likely depend on abnormalities of cortical-striatal-thalamic-cortical loops, and also the limbic system is expected to be involved [130]. Involvement of these circuits might also explain the behavioral complaints of patients with Tourette syndrome, and [^18^F]FDG PET studies show that especially hypometabolism in the (orbito-)frontal cortex was correlated with these features.

[^18^F]FDG PET studies on a wide variety of subtypes of dystonia were included in this review, resulting in many brain areas in which altered, mainly hyper-, metabolism was found. Several disease-related patterns were found, of which one, consisting of the cerebellum, basal ganglia, SMA, and midbrain, was related to both DYT-TOR1A and essential blepharospasm [44]. Also, in studies using univariate analysis, these areas were reported most often. Originally, dystonia was considered to be the result of dysfunction of the basal ganglia, but several animal and functional (imaging) studies have shown that dystonia needs to be considered a network disorder, with involvement of the brainstem, cerebellum, and motor cortex, as supported by the [^18^F]FDG PET studies [131, 132].

The studies on ataxia that were included described a wide variety of disorders with an ataxia phenotype. Despite this heterogeneity, all articles mentioned hypometabolism in the cerebellum, which is a key region in the pathophysiology of ataxia [133]. However, other regions also showed hypometabolism, including the brainstem, frontal cortex, and striatum, which was related to motor symptoms. This indicates that ataxia, similar to dystonia and other movement disorders, is a network disorder rather than solely a disorder of the cerebellum [134].

Chorea was the HMD of interest in most articles, and all studies included HD patients. Neuronal dysfunction in the striatum plays a key role in the pathophysiology of HD [135]. This is consistent with the common finding of hypometabolism in the striatum and its correlation with the severity of motor symptoms. It is important to note that in acute chorea due to a streptococcus infection, systemic lupus erythematosus, or antiphospholipid syndrome hypermetabolism of the striatum is reported in several case reports that did not meet our inclusion criteria [136–140]. Such hypermetabolism is in contrast to the hypometabolism related to chronic chorea in HD but also emphasizes that dysfunction of the striatum plays a key role in the pathophysiology of chorea. In addition, the frontal- and temporal cortex might be involved in both motor- and behavioral symptomatology of HD [135].

Studies on isolated myoclonus are limited, as most studies report on patients who also suffer from epilepsy. The resulting inclusion bias may have affected the results. Surprisingly, hypometabolism of several cortical areas was reported, indicating decreased neuronal activity. This is in contrast to the increased cortical excitability that previously has been reported in myoclonus [141]. Therefore it might be an indirect effect of epilepsy due to a metabolic exhaustion phenomenon spreading beyond the epileptogenic focus in focal seizure disorders [142]. Alterations in the thalamic glucose metabolism were frequently reported, indicating that the thalamus is another important area in the pathophysiology of myoclonus. This is supported by the occurrence of myoclonus after thalamic lesions [143].

Surprisingly, no studies on functional movement disorders were identified. However, studies using other imaging modalities, like functional MRI, have shown neural network alterations in patients with functional movement disorders [144]. Employing [^18^F]FDG PET in these patients may therefore also be a valuable addition in the search for a better understanding of this disorder.

There are a few remaining methodological issues to be taken into account when interpreting the results presented here. First, caution is necessary when interpreting the results of treatment effects on glucose metabolism in studies using only SUV data, since this method does not allow for the measurement of absolute glucose metabolism. Most studies performed an intensity normalization to a reference tissue to correct for interindividual differences. The results in these studies reflect relative changes in glucose uptake instead of absolute changes. On the other hand, such a normalization step enables the group identification of a pattern of distinct brain regions that characterizes a specific movement disorder, contributing to insight into the underlying network dysfunction. Secondly, most studies did not perform a partial volume correction, which can lead to an overestimation of hypometabolism in neurodegenerative diseases like Huntington’s [145]. For an overview of possible confounders, see Supplementary Table 5. Furthermore, the labeling of the involved regions differed between articles. To enable a visual overview of these regions, all were transformed into the AAL atlas. However, the absence of exact coordinates in most studies makes this transformation prone to subjectivity. Furthermore, most studies included a relatively small number of patients. This might have had an effect on the results, due to a lack of power. On the other hand, small sample sizes are inevitable since most HMDs are relatively rare. In order to give a fair overview, we showed significant metabolic changes in the different HMDs normalized by the number of participants in Fig. 1. Finally, the patients with HMD might have used more medications that could influence the glucose metabolism in the brain in comparison to the healthy controls. This might have influenced the results, especially in studies that included patients with epilepsy and schizophrenia.

In summary, most hyperkinetic symptomatology involved several regions and favors the possibility of network involvement. This certainly pleads for more connectivity studies using [^18^F]FDG PET for our understanding of the pathophysiology. As stated in the introduction, regional changes in cerebral metabolism may be caused not only by local neuronal impairment but also by dysfunction in remote, interconnected brain regions. The HMD-related patterns in Fig. 1 are mainly based on the results of [^18^F]FDG PET studies using a univariate analysis to compare healthy controls with patients. For some HMD, a separate network analysis was performed, and especially the network analyses performed by the Eidelberg group in dystonia, tics, and chorea yielded the most consistent disease-related patterns [9, 32, 33, 35, 37, 39, 84]. Though other functional imaging methods like fMRI were not added to the scope of this review, it is clear that fMRI may be complementary to help understand the pathophysiological underpinnings of hyperkinetic disorders.

To conclude, in all HMDs, hypo- or hypermetabolism was found in multiple, partly overlapping brain regions, and clinical characteristics often correlated with glucose metabolism. For some movement disorders, [^18^F]FDG PET metabolic changes reflected the effect of treatment.

## Supplementary Information

Below is the link to the electronic supplementary material.

Supplementary file1 (DOCX 116 KB)

## Data Availability

The datasets generated during and/or analyzed during the current study are available from the corresponding author upon reasonable request.
