# Supplementary material for: [18F]FDG PET in conditions associated with hyperkinetic movement disorders and ataxia: a systematic review
Source: Eur J Nucl Med Mol Imaging. 2023 Jan 27;50(7):1954–73. doi: 10.1007/s00259-023-06110-w (PMC10199862; doi:10.1007/s00259-023-06110-w)
Supplement: Supplementary file 1 — Supplementary file1 (DOCX 116 KB) [file 259_2023_6110_MOESM1_ESM.docx]

**Supplementary material of:**

**[^18^F]FDG** **PET in hyperkinetic movement disorders: a systematic review**

*In Scientific Reports*

Elze R. Timmers, Marrit R. Klamer, Ramesh S. Marapin, Adriaan A. Lammertsma, Bauke M. de Jong, Rudi A.J.O Dierckx, Marina A.J. Tijssen^*^

* corresponding author: prof. dr. Marina A.J. Tijssen, [m.a.j.de.koning-tijssen@umcg.nl](mailto:m.a.j.de.koning-tijssen@umcg.nl)
Department of Neurology, University Medical Center Groningen, University of Groningen, PO Box 30.001, 9700 RB Groningen, the Netherlands; Expertise Center Movement Disorders Groningen, University Medical Center Groningen (UMCG), PO Box 30.001, 9700 RB Groningen, the Netherlands

PubMed search term that was used:

(“Positron-Emission Tomography”[Mesh] OR positron emission tomograph*[tiab] OR pet*[tiab]) AND ("Fluorodeoxyglucose F18"[Mesh] OR FDG*[tiab] OR fluorodeoxyglucose*[tiab]) AND ("Movement Disorders"[Mesh] OR “Dyskinesias”[Mesh] OR "Tremor"[Mesh] OR "Ataxia"[Mesh] OR "Myoclonus"[Mesh] OR "Dystonia"[Mesh] OR "Dystonic Disorders"[Mesh] OR "Chorea"[Mesh] OR "Tics"[Mesh] OR movement disorder*[tiab] OR dyskine*[tiab] OR hyperkine*[tiab] OR tremor*[tiab] OR ataxia*[tiab] OR myoclon*[tiab] OR dystoni*[tiab] OR chorea*[tiab] OR tic*[tiab] OR tics*[tiab] OR tic disorder*[tiab] OR conversion disorder*[tiab] OR functional neurological disorder*[tiab])

| **1240** records identified from PubMed |  |  | |
| --- | --- | --- | --- |
|  |  | **0** duplicates excluded | |
|  |  |  |  |
| **1240** titles & abstracts to screen |  |  | |
|  |  | **1088**  623  271  98  35  37  22  2  0  2 | **Titles & abstracts excluded**  Hypokinetic movement disorder  Number of participants < 5  Wrong article type  Animal studies  No brain scans  Structural lesions  Other PET tracer  No English language  Other |
|  |  |  |  |
|  |  |  |  |
|  |  |  |  |
| **152** full text records to review |  |  | |
|  |  | **4** items not available for review | |
|  |  |  |  |
| **148** full text records available to review |  |  | |
|  |  | **42**  16  10  6  3  3  1  1  0  4 | Full text articles excluded  Number of participants < 5  Hypokinetic movement disorder  Wrong article type  Structural lesion  No brain scans  No English language  Other PET tracer  Animal studies  Other |
|  |  |  |  |
|  |  |  |  |
|  |  |  |  |
| **104** publications included  Reporting on **97** studies |  |  | |

Supplementary figure 1. PRISMA Flowchart

Supplementary table 1. Automatic Anatomical Labelling (AAL) atlas that was used to standardize the regions mentioned in the articles for figure 1.

| Frontal Lobe | | Parietal Lobe | |
| --- | --- | --- | --- |
| 1; 2 3; 4 5; 6 7; 8 9; 10 11; 12 13; 14 15; 16 17; 18 19; 20 21; 22 23; 24 25; 26 27; 28 69; 70 | Precentral gyrus  Superior frontal gyrus, dorsolateral  Superior frontal gyrus, orbital  Middle frontal gyrus  Middle frontal gyrus, orbital  Inferior frontal gyrus, opercular  Inferior frontal gyrus, triangular  Inferior frontal gyrus, orbital  Rolandic operculum  Supplementary motor area  Olfactory cortex  Superior frontal gyrus, medial  Superior frontal gyrus, medial orbital  Gyrus rectus  Paracentral lobule | 57; 58 59; 60 61; 62 63; 64 65; 66 67; 68 | Postcentral gyrus  Superior parietal gyrus  Inferior parietal gyrus  Supramarginal gyrus  Angular gyrus  Precuneus |
|  |  | **Central Structures** | |
|  |  | 53; 54 55; 56 57; 58 59; 60 | Caudate nucleus  Putamen  Pallidum  Thalamus |
|  |  | **Posterior Fossa** | |
|  |  | 91; 92 93; 94 95; 96 97; 98 99; 100 101; 102 103; 104 105; 106 107; 108 109 110 111 112 113 114 115 116  117  118  119 | Cerebellum crus 1 Cerebellum crus 2 Cerebellum 3 Cerebellum 4 5 Cerebellum 6 Cerebellum 7 Cerebellum 8 Cerebellum 9 Cerebellum 10 Vermis 1 2 Vermis 3 Vermis 4 5 Vermis 6 Vermis 7 Vermis 8 Vermis 9 Vermis 10 Medulla Midbrain Pons |
| Insula and Cingulate Gyri | |  |  |
| 29; 30 31; 32 33; 34 35; 36 | Insula  Cingulate gyrus, anterior part  Cingulate gyrus, mid part  Cingulate gyurs, posterior part |  |  |
| Temporal Lobe | |  |  |
| 37; 38 39; 40 41; 42 55; 56 79; 80 81;82 83; 84 85; 86 87; 88 89; 90 | Hippocampus  Parahippocampus  Amygdala  Fusiform gyrus  Heschl gyrus  Superior temporal gyrus  Temporal pole: superior temporal gyrus  Middle temporal gyrus  Temporal pole: middle temporal gyrus  Inferior temporal gyrus |  |  |
| Occipital Lobe | |  |  |
| 43; 44 45; 46 47; 48 49; 50 51; 52 53; 54 | Calcarine fissure and surrounding cortex  Cuneus  Lingual gyrus  Superior occipital lobe  Middle occipital lobe  Inferior occipital lobe |  |  |

Supplementary table 2. Overview of articles that compared results of [^18^F]DG PET scans of patients with normal controls.

| Authors (year) | Movement disorder | Outcome measure | Intensity normalization | Participants | Regions in article | Regions in AAL atlas |
| --- | --- | --- | --- | --- | --- | --- |
| Ha e.a. (2015) | Tremor | SUV | Not described | Patients with ET (n=17), and healthy controls (n=23) | temporal lobe↓, occipital lobe↓, (pre)cuneus↓, middle frontal lobe↓ | 8↓, 17↓, 23↓, 32↓, 46↓, 51↓, 56↓, 67↓, 68↓, 82↓, 85↓, 89**↓** |
| Hallett e.a. (1993) | Tremor | CMR_glu_ | Cerebral hemispheres measured at the level of basal ganglia | Patients with ET (n=8), and healthy controls (n=10) | Medulla↑, thalamus↑ | 119↑, 77↑, 78↑ |
| Schöberl e.a. (2017) | Tremor | SUV | White matter | Patients with orthostatic tremor (n=10), and healthy controls (n=10) both standing and lying | Standing vs lying: pontine tegmentum↑, posterior lobes of cerebellum↑, ventral intermediate and posterolateral nucleus of the thalamus↑, precentral gyrus↑, left paracentral gyrus↑, right inferior frontal gyrus↑, left parahippocampal gyrus↑; Lying: pontine tegmentum↑, posterior cerebellum↑, ventro intermediate and ventral posterolateral nucleus of thalamus↑, primary motor cortex↑, mesiofrontal cortical areas↓, anterior insula↓; Standing: bilateral anterior lobes and right posterior lobe of cerebellum↑, presupplementary motor area↑, anterior and posterior cingulate gyrus↓, middle temporal gyrus↓ | Standing vs. lying: 118↑, 93↑, 94↑, 99 t/m 104↑, 77↑, 78↑, 1↑, 2↑, 69↑, 12↑, 14↑, 16↑, 39↑ |
| Song e.a. (2015) | Tremor | SUV | Not described | Male patients with ET who responded to medical therapy (n=8) and those who did not (n=9), and healthy control male subjects (n=11) | Fronto-temporo-occipital lobe↓, right precuneus↓, cerebellum↓ | 3↓, 4↓, 7↓, 8↓, 15↓, 16↓, 51↓, 52↓, 68↓, 81↓, 82↓, 85↓, 86↓, 89↓, 90↓, 99↓, 100↓, 116↓ |
| Verger e.a. (2019) | Tremor | Not clearly described | Global mean | Patients with right ET (n=42) before and after left GKVIM (both responders (n=35) and non-responders (n=7)), and healthy controls (n=31) | Left thalamus↓, right cerebellum↓, left superior and middle temporal gyri↓, middle and inferior frontal gyri↓ | 77↓, 92↓, 94↓, 96↓, 98↓, 100↓, 102↓, 104↓, 106↓, 108↓, 81↓, 85↓, 7 t/m 16↓ |
| Braun e.a. (1993) | Tics | CMR_glu_ | Grey matter and average total of ROIs | Patients with Tourette syndrome (n=16), and healthy controls (n=16) | orbitofrontal↓, inferior insula↓, parahippocampal region↓, nucleus accumbens↓, ventromedial caudate↓, putamen↓, midbrain↓, premotor cortex↑, primary sensorimotor cortex↑ | 9 t/m 11↓, 15 t/m 18↓, 25↓, 29↓, 39↓, 40↓, 53↓, 73↓, 74↓, 81↓, 1↑, 2↑, 19↑, 20↑, 57↑, 58↑ |
| Eidelberg e.a. (1997) | Tics | CMR_glu_ | Global mean | Patients with Tourette syndrome (n=10), and healthy controls (n=10) | Voxel-wise and ROI: no differences, SSM: pattern I: lateral premotor and supplementary motor association cortex↑, midbrain↑; pattern II: caudatus↓, thalamus↓, lentiform nucleus↓, hippocampus↓ | pattern I: 1↑, 2↑, 19↑, 20↑, 117↑  pattern II: 71 t/m 78↓, 37↓, 38↓ |
| Pourfar e.a. (2011) | Tics | SUV | Not described | Patients with Tourette syndrome (n=12), and healthy controls (n=12) | Orbitofrontal cortex↓, striatum↓, premotor cortex↑, cerebellum↑ | 9↓, 10↓, 71 t/m74↓, 19↑, 20↑, 97 t/m 100↑ |
| Stoetter e.a. (1992) | Tics | CMR_glu_ | Global mean | Patients with Tourette syndrome (n=18), and healthy controls (n=16) | orbitofrontal cortex↓, inferior insular cortex↓, parahippocampal region↓, striatum↓, putamen↓, supplementary motor area↑, lateral premotor and Rolandic cortex↑, superior parietal lobule↑ | 5↓, 6↓, 9↓, 10↓, 11↓, 12↓, 15↓, 16↓, 29↓, 30↓, 39↓, 40↓, 71 t/m 74↓, 17 t/m 20↑, 1↑, 2↑, 59↑, 60↑ |
| Asanuma e.a. (2005) | Dystonia | CMR_glu_ | Not described | Patients with DRD (n=7), manifesting (n=11) and nonmanifesting (n=12) DYT-TOR1A carriers, manifesting (n=7) and nonmanifesting (n=6) DYT-THAP1 carriers, and healthy controls (n=14) | Dorsal midbrain↑, vermis↑, right SMA↑, (pre)motor areas↓, left putamen↓, left globus pallidus↓ | 117↑, 109 t/m 116↑, 20↑, 73↓, 75↓, 1↓,2↓, 69↓, 70↓ |
| Carbon e.a. (2004) | Dystonia | CMR_glu_ | Not described | Patients with a mutation in DYT-TOR1A both nonmanifesting (n=12) as manifesting (n=11), and in DYT-THAP1 both nonmanifesting (n=6) as manifesting (n=7), and healthy controls (n=11) | Manifesting dystonia: preSMA↑, precuneus↑, inferior parietal cortex↑; DYT-THAP1: middle and superior temporal gyrus↑, putamen↓ | 19↑, 3↑, 20↑, 67↑, 68↑, 61↑, 62↑; 86↑, 85↑ |
| Carbon e.a. (2013) | Dystonia | CMR_glu_ | Cerebral hemispheres | Patients with a mutation in DYT-SGCE, both manifesting (n=6) and nonmanifesting (n=6), DYT-TOR1A (n=18), DYT-THAP1 (n=13), DRD (n=9), posthypoxic myoclonus (n=7) and healthy controls (n=24) | DYT-SGCE: Ventral inferior pons↑, right posterior thalamus↑, ventromedial prefrontal cortex↓, left lobule V of cerebellum (only manifesting)↑; all dystonia subtypes: superior parietal lobule ↑; DYT-TOR1A: ventromedial prefrontal cortex↓ | 118↑, 78↑, 24↓, 23↓; 97↓; 61↑; 24↓ |
| Chase e.a. (1988) | Dystonia | CMR_glu_ | Not described | Patients with idiopathic torsion dystonia (n=6), and healthy controls (n=9) | Contralateral lenticular nucleus↑ | 73 t/m 76↑ |
| Eidelberg e.a. (1995) | Dystonia | CMR_glu_ | Global mean | Patients with idiopathic torsion dystonia (n=11), and healthy controls (n=11) | ROI analysis: no differences, SSM analysis: lateral premotor region↑, SMA↑, left lentiform nucleus↑, pons↑, midbrain↑ | 69↑, 70↑, 19↑, 20↑, 73↑, 75↑, 117↑, 118↑ |
| Eidelberg e.a. (1998) | Dystonia | CMR_glu_ | Not described | Patients with DYT-TOR1A dystonia both manifesting (n=10) and non-manifesting(n=7), and healthy controls (n=14) | Non-manifesting (also present in manifesting): cerebellum↑, caudate nucleus↑, lentiform nucleus↑, SMA↑, midbrain↓ | 91 t/m 116↑, 71 t/m 76↑, 19↑, 20↑, 117↓ |
| Galardi e.a. (1996) | Dystonia | CMR_glu_ | Not described | Patients with spasmodic torticollis (n=10) and healthy controls (n=15) | Anterior and middle cingulate cortices↑, lenticular nucleus↑, thalamus↑, cerebellum↑ | 31 t/m 34↑, 73 t/m 78↑, 91 t/m 116↑ |
| Hutchinson e.a. (2000) | Dystonia | CMR_glu_ | Not described | Patients with essential blepharospasm (n=6), and healthy controls (n=6) | Wakefulness: cerebellum↑, pons↑; Sleep: superior-medial premotor frontal cortex↓ | 91 t/m 116↑, 118↑ |
| Lalli e.a. (2012) | Dystonia | SUV | Global mean | Patients with cervical dystonia (n=5) and healthy controls (n=?) | pre- and postcentral gyrus↑ | 1↑, 2↑, 57↑, 58↑ |
| Liu e.a. (2021) | Dystonia | SUV | Global mean | Patients with Meige syndrome (n=50) untreated and right handed, and healthy controls (n=50) | Left internal globus pallidus↓, left parietal lobe↓, right frontal lobe↓, right postcentral gyrus↓, thalamus↓, cerebellum↓ | 75↓, 69↓, 17↓, 58↓, 77↓, 78↓, 91 t/m 116↓ |
| Martin e.a. (1988) | Dystonia | CMR_glu_ | Not described | Patients with idiopathic torticollis (n=16), and healthy controls (n=11) | No significant differences | - |
| Otsuka e.a. (1992) | Dystonia | CMR_glu_ | Not described | Patients with idiopathic dystonia (n=8) subdivided into generalized dystonia (n=3), focal dystonia in the arm (n=3), cervical dystonia (n=2) and healthy controls (n=8) | No significant differences | - |
| Suzuki e.a. (2007) | Dystonia | SUV | Global mean | Patients with essential blepharospasm (n=25), and healthy controls (n=38) | Thalamus↑, pons↑ | 77↑, 78↑, 118↑ |
| Suzuki e.a. (2014) | Dystonia | SUV | Not described | Patients with drug induced blepharospasm (n=21) and essential blepharospasm (n=21), healthy subjects using benzodiazepines (n=24), and healthy controls (n=63) | Drug induced blepharospasm: thalamus↑; Essential blepharospasm: thalamus↑, putamen↑ | 77↑, 78↑; 77↑, 78↑, 73↑, 74↑ |
| Suzuki e.a. (2019) | Dystonia | SUV | Global mean | Patients with essential blepharospasm (n=39), healthy controls with closed eyes (n=48) and open eyes (n=48) | Patients vs eye open: posterior and anterior extrastriate cortex↓, thalamus↑; Patients vs eyes closed: thalamus↑ | 77↑, 78↑, 53↓, 54↓ |
| Aguiar e.a. (2016) | Ataxia | SUV | Global mean | Patients with SCA36 (n=20), and healthy controls (n=78) | Cerebellar hemispheres (r > l) ↓, vermis ↓, brainstem ↓ | 91 t/m 108↓, 109 t/m 116↓, 117 t/m 119↓ |
| Brockmann e.a. (2012) | Ataxia | SUV | Cortical grey matter (excluding region with hypometabolism) | Patients with SCA17, both manifesting (n=5) and nonmanifesting (n=4), and healthy controls (n=9) | Caudate nucleus ↓, putamen ↓, cingulum↓, parietal cortex↓, (pre)cuneate region↓ | 71 t/m74↓, 31 t/m 36↓, 61↓, 45, 46↓ |
| Gilman e.a. (1988) | Ataxia | CMR_glu_ | Not described | Patients with OPCA (n=30), and healthy controls (n=30) | Cerebellar hemispheres↓, vermis↓, brainstem↓ | 91 t/m 116↓, 117 t/m 119↓ |
| Gilman e.a. (1994) | Ataxia | CMR_glu_ | 1. No normalization 2. Cerebral cortex | Patients with sporadic OPCA (n=32), dominantly inherited OPCA (n=19), MSA (n=24), and healthy controls (n=51) | All OPCA patients: cerebellum↓, brainstem↓ | 91 t/m 116↓, 117↓, 118↓ |
| Gilman e.a. (1995) | Ataxia | CMR_glu_ | Cerebral cortex | Patients with OPCA (n=17), and healthy controls (n=21) | Cerebellar vermis↓, brainstem↓, thalamus↓ | 109 t/m 116↓, 117↓, 118↓, 77↓, 78↓ |
| Grimaldi e.a. (2019) | Ataxia | SUV | Global mean | Patients with MSA (n=58) subdivided into patients with extrapyramidal, axial, laryngeal-pharyngeal involvement (LPI) and cerebellar symptoms (n = 46)(1), cerebellar and LPI symptoms (n = 30)(2) and cerebellar and cognitive symptoms (n = 9)(3), and healthy controls (n=?) | Profile 2: medulla↓, prefrontal-↓, temporal-↓, cingulate cortex↓, putamen↓, cerebellar hemispheres↓; profile 3: posterior cerebellar hemispheres↓, vermis↓ | Profile 2: 119↓, 3↓, 4↓, 7↓, 8↓ ,11 t/m 14↓, 31↓, 33 t/m36↓, 73↓, 74↓, 81↓, 82↓, 85↓, 86↓, 89 t/m108↓; profile 3: 91 t/m 94↓, 109 t/m116↓, 101↓, 102↓ |
| Lee e.a. (2008) | Ataxia | SUV | Not described | Patients with MSA-C (n=41), and healthy controls (n=30) | Cerebellum ↓, brainstem↓, frontal cortex area↓, cingulum↓, parietal cortex↓ | 91 t/m 116↓, 3↓, 4↓, 7↓, 15↓, 16↓, 23↓, 27↓, 28↓, 31↓, 32↓, 62↓, 117 t/m 119↓ |
| Matthew e.a. (1993) | Ataxia | CMR_glu_ | Grey matter | Patients with OPCA (n=14) subdivided in affected (n=7) and non-affected (n=7), and healthy controls (n=43) | Cerebellar hemispheres↓, vermis↓, brainstem↓, frontal and prefrontal areas↓, temporal areas↑, left parietal/occipital region↑ | 91 t/m 108↓, 109 t/m 116↓, 3↓,4↓,7↓,8↓,12↓,13↓,14↓,23↓,24↓ 49↑,61↑,80↑,82↑, 85↑, 86↑, 117 t/m119↓ |
| Meles e.a. (2018) | Ataxia | SUV | Global mean | Patients with SCA3 (n=17), and healthy controls (n=16) | Cerebellum↓, caudate nucleus↓, posterior parietal cortex↓, somatosensory areas↑, limbic system↑ | 91 t/m 116↓, 71 t/m 74↓, 67↓, 68↓, 62↓, 59↑, 60↑, 29↑, 30↑, 31 t/m 34↑, 37 t/m 42↑, 9↑, 10↑, 119↓, 117↓ cerebellar peduncles↑ |
| Mishina e.a. (1999) | Ataxia | SUV | Global mean | Patients with OPCA in early phase (n=9), and healthy controls (n=10) | Activation ratio (walking/resting) in pyramis↓ | 119↓ |
| Oh e.a. (2014) | Ataxia | SUV | Not described | Patients with SCA (n=12), and healthy controls (n=12) | Whole cerebellum↓, anterior/posterior ratio↓ | 91 t/m 116↓ |
| Oh e.a. (2017) | Ataxia | SUV | Occipital cortex | Patients with multi system atrophy of the cerebellar type (n=44), SCA2 (n=9), SCA6 (n=14) and crossed cerebellar diaschisis (n=15), and healthy controls (n=89) | MSA-C: whole cerebellum↓ SCA2: whole cerebellum↓ SCA6: whole cerebellum except inferior posterior vermis↓ | MSA-C: 91 t/m 116↓, SCA2: 91 t/m116↓, SCA6: 91 t/m 114↓ |
| Otsuka e.a. (1994) | Ataxia | CMR_glu_ | Occipital cortex | Patients with progressive ataxia (n=11) subdivided into cerebellar cortical degeneration (n=5 (late cerebellar cortical atrophy (n=3), Holmes type hereditary ataxia (n=2)), olivopontocerebellar atrophy (n=6), and healthy controls (n=16) | OPCA: cerebellum↓, brainstem↓, caudate nucleus↓, putamen↓; CCD: cerebellum↓ | OPCA: 91 t/m 116↓, 71 t/m 74↓, 117 t/m 119↓; CCD: 91 t/m 116↓ |
| Rudolf e.a. (2000) | Ataxia | CMR_glu_ | Global mean | Patients with inherited or idiopathic cerebellar ataxia (n=23) subdivided into early onset (n=8), late onset (n=9), onset >50 y (n=6), and healthy controls (n=16) | EOCA: cerebellar hemispheres↓, vermis↓, dentate nucleus↓, thalamus↓, pons↓; LOCA: cerebellar hemispheres↓, dentate nuclei↓, (pre)cuneus↑, gyrus supramarginalis↑; onset>50 years: cuneus↑; All groups only pure ataxia: pontine and brainstem regions↓ | EOCA: 91 t/m 116↓, 77↓, 78↓, dentate nucleus↓, 118↓; LOCA: 91 t/m 108↓, 67↑, 68↑, 45↑, 46↑, 63↑, 64↑, dentate nucleus↑; onset>50 yrs: 45↑, 46↑, all patients: 117 t/m 119↓ |
| Soong e.a. (2001) | Ataxia | CMR_glu_ | Not described | Patients with SCA6 (n=7), and healthy controls (n=7) | Cerebellar hemispheres↓, brainstem↓, basal ganglia↓, frontal -, temporal- and occipital cortex ↓ | 91 t/m 108↓, 71 t/m 76↓, 3 t/m 16↓, 23 t/m 26↓, 81 t/m 90↓, 49 t/m 54↓, 117 t/m 119↓ |
| Volkow e.a. (2014) | Ataxia | CMR_glu_ | 1. No normalization 2. Global mean | Patients with ataxia-telangiectasia (n=10), non-affected relatives (n=19), and healthy controls (n=29) | Cerebellar hemispheres(crus I and II)↓, anterior vermis (I-IV, IX)↓, fusiform gyrus↓, hippocampus↓, globus pallidus↑, vermis (VI)↑ | 37↓, 38↓, 55↓, 56↓, 91 t/m 94↓, 109↓, 110↓, 111↓, 115↓, 112↑, 75↑, 76↑ |
| Wang e.a. (2007) | Ataxia | Not described | Global mean | Patients with SCA2 (n=8), SCA3 (n=12), SCA6 (n=7), sporadic olivopontocerebellar ataxia (n=3), and healthy controls (n=24) | SCA: cerebellum↓, thalamus↓ and caudate nucleus↓ | SCA patients: 91 t/m 116↓, 77↓, 78↓, 71↓, 72↓ |
| Wang e.a. (2007) | Ataxia | Not described | Global mean | Patients with SCA2 (n=9), SCA3 (n=12), SCA6 (n=7), and healthy controls (n=23) | SCA2: cerebellum↓, pons↓, parahippocampal gyrus↓, frontal cortex↓, left hippocampus↓; SCA3: cerebellum↓, parahippocampal gyrus↓, right lentiform nucleus↓; SCA6: cerebellum↓, (pre)frontal cortex↓ | SCA2: 91 t/m 116↓, 39↓, 40↓, 37↓, 15↓,2↓, 8↓, 118↓, SCA3: 97↓, 98↓, 99↓, 100↓, 111↓, 39↓, 40↓, 74↓, 76↓; SCA6: 91 t/m 116↓, 1↓, 2↓, 57↓, 4↓ |
| Wüllner e.a. (2005) | Ataxia | Not described | Global mean excluding cerebellum, diencephalon, brainstem and striatum | Patients with SCA1 (n=5), SCA2 (n=4), SCA3 (n=6), SCA6 (n=6), Parkinson's disease (n=10), and healthy controls (n=10) | SCA1: cerebellum↓, brainstem↓; SCA2: cerebellum↓, brainstem↓, parietal cortex↓; SCA3: cerebellum↓, brainstem↓, thalamus↓, temporal lobe↑; SCA6: cerebellum↓, temporal lobe↑ | SCA1: 91 t/m 116↓, 117 t/m 119↓; SCA2: 91 t/m 116↓, 59 t/m 62↓, 117 t/m 199↓; SCA3: 91 t/m 116↓, 77↓, 78↓, 117 t/m 119↓, 84↑, 85↑, 86↑, 88↑, 90↑; SCA6: 91 t/m116↓, 83 t/m 90↑ |
| Allain e.a. (2011) | Chorea | SUV | Not described | Patients with early stage HD (n=8), and healthy controls (n=12/9) | Caudate nucleus ↓, putamen ↓, prefrontal-↓, anterior cingulate cortex↓, parietal lobe↓, sensimotor and premotor regions↓ | 71 t/m 74↓, 1 t/m 8↓, 19↓, 20↓, 23↓, 24↓, 31↓, 32↓, 57↓, 58↓, 61↓, 62↓ |
| Antonini e.a. (1996) | Chorea | CMR_glu_ | Mean value of plain in which ROI was defined | Patients with HD (n=8) and asymptomatic gene carriers (n=10) and healthy controls (n=20) | Caudate nucleus↓, putamen↓ | 71 t/m 74↓ |
| Berent e.a. (1988) | Chorea | CMR_glu_ | Cortical value of plane in which ROI was defined. | Patients with early to mid-stage HD (n=15) and healthy controls (n=14) | Caudate nucleus↓, putamen↓ | 71 t/m 74↓ |
| Ciarmiello e.a. (2006) | Chorea | SUV | Global mean | Patients with HD (n=71) subdivided into presymptomatic (n=24) and advanced cases (n=47), and healthy controls (n=30) | Frontal and temporal cortex↓, striatum↓ | 71 t/m 74↓, 81↓, 82↓, 85↓, 86↓, 89↓, 90↓, 3 t/m 16↓ |
| Feigin e.a. (2001) | Chorea | CMR_glu_ | Global mean | Patients with early stage HD (n=13), asymptomatic gene carriers (n=18), and healthy controls (n=8) | Caudate nucleus↓, putamen↓ | 71 t/m 74↓ |
| Kuhl e.a. (1984) | Chorea | CMR_glu_ | Cortical values of plane in which ROI was defined | Patients with manifest (n=13) and at risk for HD (n=15), patients with Parkinson's disease (n=8), and healthy controls (n=13) | Caudate nucleus↓, putamen↓ | 71 t/m 74↓ |
| Kuwert e.a. (1992) | Chorea | CMR_glu_ | Not described | Patients with early HD (n=12), and healthy controls (n=12) | Caudate nucleus↓ | 71↓, 72↓ |
| Kuwert e.a. (1993) | Chorea | CMR_glu_ | Cerebellum | Patients with manifest (n=6) and pre-manifest HD (n=12), and healthy controls (n=20) | Caudate nucleus↓, lentiform nucleus↓ | 71 t/m 76↓ |
| Kuwert e.a. (1993) | Chorea | CMR_glu_ | Cerebellum | Patients with manifest (n=20) and pre-manifest HD (n=27), and healthy controls (n=20) | Caudate nucleus↓, lentiform nucleus↓ | 71 t/m 76↓ |
| Lee e.a. (2012) | Chorea | SUV | 1. Global mean 2. Pons 3. Cerebellum 4. White matter 5. Thalamus 6. Pons-cerebellar vermis | Patients with manifest HD (n=11), and healthy controls (n=11) | white matter as intensity normalization: Caudate nucleus↓, putamen↓, pallidum↓, superior and middle frontal gyri↓, superior and inferior parietal gyri↓, right postcentral gyri↓, left precentral gyrus↓, thalamus↑, inferior occipital↑, rolandic operculum↑, supramarginal gyri↑, calcarine↑, right lingual↑ | 71 t/m 76↓, 1↓, 3↓, 4↓, 7↓, 8↓, 58 t/m 62↓, 17↑, 18↑, 20↑, 43↑, 44↑, 48↑, 53↑, 54↑ |
| López-Mora e.a. (2016) | Chorea | SUV | Pons | Patients with manifest (n=18) and pre-manifest HD (n=15), and healthy controls (n=18) | Striatum↓, prefrontal and temporal cortex↓ | 71 t/m 74↓, 3 t/m 16↓, 81 t/m 90↓ |
| Martin e.a. (1992) | Chorea | CMR_glu_ | Not described | Patients with HD (n=39), and healthy controls (n=34) | Striatum↓, thalamus↓, all cortical areas except temporal ↓ | 71 t/m 74↓, 77↓, 78↓, 61↓, 62↓, 7↓, 8↓, 13 t/m 16↓, 23↓, 24↓ |
| Martin e.a. (1995) | Chorea | CMR_glu_ | 1. Thalamus 2. Mean value of plane in which ROI was defined | Patients with early stage HD (n=6), and healthy controls (n=33) | Caudate nucleus↓ | 71↓, 72↓ |
| Mayberg e.a. (1992) | Chorea | CMR_glu_ | Grey matter | Patients with early stage HD (n=9) subdivided into depressed (n=4) and non-depressed patients (n=5), and healthy controls (n=7) | Non-depressed: caudate nucleus↓, putamen↓, cingulate gyrus↓; Depressed patients only: orbitofrontal cortex↓ and inferior prefrontal cortex↓, caudate nucleus↓, putamen↓, cingulate gyrus↓ | 71 t/m 74↓, 31 t/m 36↓; 71 t/m 74↓, 31 t/m 36↓, 5↓,6↓, 9↓, 10↓, 11 t/m 16↓, 25↓, 26↓ |
| Mazziotta e.a. (1987) | Chorea | CMR_glu_ | Cerebral hemispheres, limited to supratentorial regions | Asymptomatic patients at risk for HD (n=58), symptomatic patients with HD (n=10), and healthy controls (n=27) | Caudate nucleus↓ | 71↓, 72↓ |
| Otsuka e.a. (1993) | Chorea | CMR_glu_ | Cerebellum | Patients with chorea (n=12, subdivided into with dementia (n=7) and without (n=5)): HD (n=5), sporadic chorea with dementia without family history (n=2), choreoacanthocytosis (n=2), hemichorea caused by vascular lesions (n=2), and healthy controls (n=16) | All patients: Caudate nucleus↓, putamen↓; patients with dementia: frontal-, temporal and parietal cortex↓ | 71 t/m 74↓; 3 t/m 16↓, 59 t/m 62↓, 81↓, 82↓, 85↓, 86↓, 89↓, 90↓ |
| Young e.a. (1986) | Chorea | CMR_glu_ | Peak cortical activity of plane in which ROI was defined | Drug-free patients with early to midstage HD (n=15), and healthy controls (n=14) | All patients: caudate nucleus↓, putamen↓, thalamus↑ | 71 t/m 74↓, 77↑, 78↑ |
| Young e.a. (1987) | Chorea | CMR_glu_ | Cortex | Persons at risk for HD (n=29), patients with stage I (n=9), and patients with stage II (n=8) symptomatic HD, and healthy controls (n=28) | Stage I and II HD patients: caudate nucleus↓ | 71↓, 72↓ |
| Berkovic e.a. (1989) | Myoclonus | CMR_glu_ | Cerebral cortex in the temporo-occipital area | Patients with myoclonus epilepsy and ragged red fibers (n=5) and healthy controls (n=6) | Frontal-, central-, parietal-, temporal-, occipital cortex↓, basal ganglia↓, thalamus↓, vermis↓, brainstem↓, corona radiata↓ | 1 t/m 16↓, 57 t/m 62↓, 69 t/m 78↓, 49 t/m 54↓, 81 t/m 90↓, 109 t/m 119↓ |
| Frucht e.a. (2004) | Myoclonus | CMR_glu_ using a simplified aterial input function | Global mean | Patients with myoclonus after cardiac arrest without significant atrophy or focal neurological deficits (n=7), and healthy controls (n=10) | Ventral lateral thalamus↑ , pontine tegmentum↑, medial temporal lobes↑ | 77↑, 78↑, 118↑ |
| Kim e.a. (2005) | Myoclonus | SUV | Global mean | Patients with juvenile myoclonic epilepsy (n=19), and healthy controls (n=19) | Thalamus↑ | 77↑, 78↑ |
| McDonald e.a. (2006) | Myoclonus | CMR_glu_ | Global mean | Patients with juvenile myoclonus epilepsy (n=10), frontal lobe epilepsy (n=18) and healthy controls (n=14) | No differences | x |
| Muccioli e.a. (2020) | Myoclonus | SUV | Not described | Patients with Lafora disease (n=8), almost all with myoclonus and ataxia (n=7), and healthy controls (n=?) | Temporal-, parietal- and frontal lobe↓, thalamus↓ | 3 t/m 16↓, 81 t/m 90↓, 59 t/m 62↓, 77↓, 78↓ |
| Swartz e.a. (1996) | Myoclonus | CMR_glu_ | Global mean | Patients with Juvenile myoclonus epilepsy (n=9), and healthy controls (n=14) | Left caudate nucleus↓, left premotor area↓ | 71↓, 19↓ |
| Dubroff e.a. (2008) | Metabolic disease | SUV | Not described | Patients with galactosaemia (n=5), and healthy controls (n=8) | Superior temporal gyrus↓, medial occipital lobe↓, cerebellum↓, calcerine cortex↓, superior frontal cortex↓, superior parietal cortex↓, caudate↓, precentral gyrus↓, cingulate gyrus↑, temporal poles↑, subcallosal gyrus↑, claustrum↑ | 81↓, 82↓, 51↓, 52↓, 59↓, 60↓, 109 t/m 116↓, 43↓, 44↓, 3↓, 4↓, 71↓, 72↓, 1↓, 2↓, 31 t/m 36↑, 83↑, 84↑, 87↑, 88↑ , subcallosal gyrus↑, claustrum↑ |
| Hermann e.a. (2002) | Metabolic disease | CMR_glu_ | Not described | Patients with Wilson's disease (n=37), and healthy controls (n=9) | Caudate nucleus↓ | 71↓, 72↓ |
| Kuwert e.a. (1992) | Metabolic disease | CMR_glu_ | Not described | Patients with Wilson's disease (n=14), and healthy controls (n=23) | Cerebellum↓, caudatus↓, cortex↓, thalamus↓, lentiform nucleus↓ | 91 t/m 116↓, 71 t/m 78↓, 3 t/m 16↓, 49 t/m 54↓, 59 t/m 62↓, 81↓, 82↓, 85↓, 86↓, 88↓, 89↓ |
| Lau e.a. (2021) | Metabolic disease | SUV | Not described | Patients with Niemann-Pick disease type C (n=14), and healthy controls (n=33) | Frontal lobe↓, thalamus↓, posterior cingulate gyrus↓ | 77↓, 78↓, 3 t/m 10↓, 31↓, 32↓, 35↓, 36↓ |
| Schlaug e.a. (1996) | Metabolic disease | CMR_glu_ | Global mean | Patients with Wilson's disease (n=18), and healthy controls (n=17) | Severely affected: striatum↓, cerebellum↓, thalamus↓, cerebral cortex↓ | 71 t/m 74↓, 77↓, 78↓, 91 t/m 116↓, 3 t/m 16↓, 49 t/m 54↓, 59 t/m62↓, 81↓, 82↓, 85↓, 86↓, 89↓, 90↓ |
| Suhonen-Polvi e.a. (1999) | Metabolic disease | CMR_glu_ | Not described | Patients with Salla disease (n=9), four control groups: infants < 1 y with suspected hypoxic-ischemic brain injury (n=12), children 1-2 y and 3-8 y with transient neurologic disorders (n=16), children 9-17 y with migraine without daily medication (n=9), healthy male medical students 24-25 years old (n=7) | Basal ganglia↑, frontal and sensorimotor cortex↑; ataxic patients: cerebellum↓ | 71 t/m 76↑, 1 t/m 16↑, 57↑, 58↑; 91 t/m 116↓ |
| Kerik- Rotenberg e.a. (2020) | Auto-  immune | SUV | Global mean | Patients with NMDA-ab encephalitis (n=33) of which 58% had a movement disorder (n=19), hyperkinetic (n=6)/mixed(n=17)/hypokinetic(n=3), and healthy controls (n=14) | Cerebellum (peduncle and VIII)↑, temporal lobe (right pole and amygdala)↑, right insula↑, occipital-↓, parietal lobe↓, Heschl's gyrus↓ | 103↑, 104↑, 84↑, 88↑, 42↑, 30↑, 35↓, 36↓, 43 t/m 54↓, 57↓, 58↓, 65 t/m 68↓, 79↓, 80↓ |
| Kim e.a. (2009) | Auto-  immune | SUV | Global mean | Patients with Fisher's syndrome (n=10), and healthy controls (n=60) | Cerebellar hemispheres↑, vermis↑, pontine tegmentum↑, midbrain tectum↑, right inferior frontal cortex↑, left thalamus↑, occipital cortex↓ | 91 t/m 108↑, 109 t/m 116↑, 117↑, 118↑, 16↑, 77↑, 51↓, 52↓ |
| Wang e.a. (2021) | Auto-  immune | SUV | Not described | Patients with stiff person syndrome (n=22, subdivided into classic (n=16), and variant (n=6)) or cerebellar ataxia (n=8) associated with anti-GAD65 antibodies, and healthy controls (n=50) | Classic stiff person syndrome: right superior lateral temporal cortex↑, right posterior medial temporal cortex↑, right anterior medial temporal cortex↑, right caudate nucleus↑, associative visual cortex↑, pons↑, right inferior lateral posterior temporal cortex↓, right inferior frontal cortex↓, left thalamus↓, left posterior cingulate cortex↓, left medial frontal cortex↓, left inferior frontal cortex↓, left area of Broca↓; cerebellar ataxia: vermis↑, right superior lateral temporal cortex↑, right posterior medial temporal cortex↑, right anterior medial temporal cortex↑, cerebellum↑, pons↑, midbrain↑, right inferior frontal cortex↓, left posterior cingulate cortex↓, left mid frontal cortex↓, left broca area↓; variant: right superior lateral temporal cortex↑, right posterior medial temporal cortex↑, midbrain↑ | 82↑, 86↑, 72↑, 51↑, 52↑, 118↑, 90↑, 12↓, 14↓, 16↓, 77↓, 35↓, 7↓; 109 t/m 116↑, 82↑, 86↑, 91 t/m 108↑, 118↑, 117↑, 12↓, 14↓, 16↓, 49↓, 7↓, 13↓; 82↑, 86↑, 117↑ |
| Zhao e.a. (2021) | Auto-  immune | SUV | Cerebellum | Patients with LGI1-ab encephalitis (n=25) of which 36% present with FBDS (n=9), and healthy controls (n=44) | Only patients with FBDS: putamen↑ | 73↑, 74↑ |
|  |  |  |  |  |  |  |

Regions in which hypermetabolism (↑) or hypometabolism (↓) was found are given as stated in the article and as regions in the Automatic Anatomical Labeling (AAL) atlas. Numbers correspond to numbers in the AAL atlas (see supplementary table 1). Results of visual analysis are not shown in figure 1. The following abbreviations are used: SUV: standardized uptake value; CMR_glu_: cerebral metabolic rate of glucose; ROI: region of interest; OPCA: olivopontocerebellar atrophy; SCA: spinocerebellar ataxia; MSA(-C): multi system atrophy (of the cerebellar type); LG1I-ab encephalitis: leucine rich, glioma inactivated 1, antibody encephalitis; FBDS: facio-brachial dystonic seizures; NMDA-ab encephalitis: N-methyl-D-aspartate receptor antibody encephalitis; HD: Huntington’s disease; ET: essential tremor; GKVIM: Gamma knife of left ventral intermediate nucleus; (pre)SMA: (pre)supplementary motor area.

Supplementary table 3. Overview of articles that compared results of ^18^FDG PET scans of patients with another control group than healthy controls.

| Authors (year) | Movement disorder | Outcome measure | Intensity normalization | Comparison | Results |
| --- | --- | --- | --- | --- | --- |
| Reich e.a. (2016) | Tremor | SUV | Global mean | Patients with ET treated with bilateral thalamic DBS who developed gait ataxia (n=5) vs. those who did not (n=5) | Cerebellum ↑ |
| Sun e.a. (2019) | Tremor | SUV | Cerebellum | 1. ET (n=8) vs. MSA patients (n=10); 2. ET vs. patients with parkinsonism (n=38) | 1. Pons↑, cerebellum↑; 2. No significant differences |
| Asanuma e.a. (2005) | Dystonia | CMR_glu_ | Not described | Patients with DRD (n=7) vs. DYT-TOR1A carriers (n=23), DYT-THAP1 carriers (n=13) | DRD-related pattern (Dorsal midbrain↑, vermis↑, right SMA↑, (pre)motor areas↓, left putamen↓, left globus pallidus↓): significantly lower subject scores in DYT-TOR1A and DYT-THAP1 patients |
| Belenky e.a. (2018) | Dystonia | SUV | Global mean | Patients with focal or segmental dystonia (n=10) vs. ET(n=4) | Left thalamus↓, right nucleus lenticularis↓, gyrus cingularis↑, pons↑ |
| Carbon e.a. (2004) | Dystonia | CMR_glu_ | Not described | 1. Patients with a mutation in DYT-TOR1A both nonmanifesting (n=12) vs. manifesting (n=11); 2. Patients with DYT-TOR1A vs. DYT-THAP1 (n=13); 3. Patients with DYT-THAP1 vs. DYT-TOR1A | 1. Superior frontal gyrus↑, preSMA↑, precuneus↑, inferior parietal cortex↑; 2. Inferior cerebellum↑, putamen↑, anterior cingulate cortex↓; 3. Middle and superior temporal gyrus↑ |
| Carbon e.a. (2013) | Dystonia | CMR_glu_ | Cerebral hemispheres | Patients with a mutation in DYT-SGCE, manifesting (n=6) vs. nonmanifesting (n=6) | Left parasagittal cerebellum↑ |
| Eidelberg e.a. (1998) | Dystonia | CMR_glu_ | Not described | Patients with DYT-TOR1A dystonia, manifesting (n=10) vs. non-manifesting (n=7) | Cerebellum↑, midbrain↑, thalamus↑, lateral premotor cortex↑, SMA↑ |
| Esmaeli-Gutstein e.a. (1999) | Dystonia | Not described | Mean value of plane in which ROI was defined | Patients with essential blepharospasm (n=10), comparison within patients compared to other regions | Striatum↑, thalamus↑ |
| Suzuki e.a. (2014) | Dystonia | SUV | Not described | Patients with drug induced blepharospasm (n=21) vs. essential blepharospasm (n=21) | Thalamus↓ |
| Szymanski e.a. (1996) | Dystonia | CMR_glu_ | Global mean | Schizophrenic patients who developed tardive dyskinesia (n=8) vs. those who did not (n=8) | Temporal limbic area↑, brainstem↑, cerebellum↑, parietal- and cingulate gyrus↓ |
| Szyszko e.a. (2015) | Dystonia | SUV | Global mean | Patients with NBIA vs. primary dystonia subdivided into DYT-TOR1A (n=2), DYT-SGCE (n=2) and a group without genetic diagnosis (n=12) | Posterior cingulate cortex↑, corpus callosum↑, posterior putamina↑, lingual and fusiform gyrus of occipital cortex↓, cerebellar crusiform gyrus↓, anterior cingulate cortex↓, left insular cortex↓ |
| Gilman e.a. (1988) | Ataxia | CMR_glu_ | Cerebral cortex | Patients with sporadic OPCA (n=14) vs. familial cases (n=16) | Vermis↓, cerebellar hemispheres↓ |
| Grimaldi e.a. (2019) | Ataxia | SUV | Global mean | Patients with MSA (n=58) subdivided into patients with extrapyramidal, axial, laryngeal-pharyngeal involvement (LPI) and cerebellar symptoms (n = 46)(1), cerebellar and LPI symptoms (n = 30)(2) and cerebellar and cognitive symptoms (n = 9)(3)   1. Profile 1 vs. 2; 2. Profile 1 vs. 3; 3. Profile 2 vs. 3 | 1. Lenticular-↓, putaminal nuclei↓, anterior cerebellum↑; 2. Frontal gyrus↓, posterior cerebellum↑; 3. Frontal gyrus ↓ |
| Lee e.a. (2008) | Ataxia | SUV | Not described | Patients with MSA of the cerebellar type with a UMSARS < 30 (n=14) vs. patients with UMSARS ≥ 60 (n=14) | Left inferior frontal cortex↓, right inferior orbitofrontal lobe↓, right anterior and middle cingulate gyrus↓, anterior portion of superior mesiofrontal gyrus↓, dorsal midbrain↓ |
| Matthew e.a. (1993) | Ataxia | CMR_glu_ | Grey matter | Affected patients with OPCA (n=7) vs. non-affected (n=7) | Overall global metabolic rate ↓, cerebellar hemispheres↓, vermis↓, brainstem↓, frontal and prefrontal areas↓, left middle temporal area↑ |
| Oh e.a. (2017) | Ataxia | SUV | Not described | 1. Patients with SCA2 (n=9) vs. MSA-C (n=44); 2. Patients with SCA6 (n=14) vs. MSA-C; 3. Patients with SCA6 vs. SCA2 | 1. Right dentate nucleus↓, right anterior cortex of cerebellum↓; 2. Superior posterior cortices and left middle posterior cortex and left inferior posterior cortex of the cerebellum↑, inferior posterior vermis↑, anterior vermis↓; 3. Superior, middle and inferior posterior cortices of the cerebellum↑ |
| Rudolf e.a. (2000) | Ataxia | CMR_glu_ | Global mean | Patients with inherited or idiopathic cerebellar ataxia with   1. early onset (n=8) vs. late onset (n=9); 2. Early onset vs. onset >50 yrs (n=6); 3. Late onset vs. onset >50 yrs | 1. Thalamus↓; 2. Thalamus↓; 3. Cuneus↑ |
| Volkow e.a. (2014) | Ataxia | CMR_glu_ | 1. None 2. Global mean | 1. Patients with ataxia-telangiectasia (n=10) vs. non-affected relatives (n=19); 2. Relatives vs. healthy controls (n=29) | 1. Global mean normalization: Cerebellar hemispheres↓, anterior vermis↓, fusiform gyrus↓; 2. Global mean normalization: Anterior vermis↓, hippocampus↓ |
| Wang e.a. (2007) | Ataxia | Not described | Global mean | 1. Patients with SCA2 (n=9) vs. SCA3 (n=12); 2. Patients with SCA2 vs. SCA6 (n=7); 3. Patients with SCA3 vs. SCA6 | 1. Cerebellum↓, lentiform nucleus↑, midbrain↑; 2. Pons↓, cerebellum↓; 3. Pons↓, medulla↓ |
| Zhao e.a. (2020) | Ataxia | SUV | Not described | Patients with MSA-C (n=13) vs. unknown | Cerebellum↓, medulla oblongata↓, parental and frontal lobe↓ |
| Antonini e.a. (1996) | Chorea | CMR_glu_ | Mean value of plane in which ROI was defined | Patients with HD (n=8) vs. pre-manifest gene carriers (n=10) | Caudate nucleus↓, putamen↓ |
| Ciarmiello e.a. (2006) | Chorea | SUV | Global mean | Patients with HD (n=21) PET scan at 0 months vs. 12 months later | Frontal-, temporal and parietal lobe↓, caudate nucleus↓, putamen↓ |
| López-Mora e.a. (2016) | Chorea | SUV | Pons | Patients with HD (n=18) vs. pre-manifest gene carriers (n=15) | Striatum↓ |
| López-Mora e.a. (2019) | Chorea | SUV | 1. Global mean 2. Pons 3. Cerebellum 4. White matter 5. Thalamus 6. Pons-cerebellar vermis | Patients with HD (n=38) vs. pre-manifest gene carriers (n=20) | White matter normalization: Cerebellum↑, pons↑, thalamus↑, parietal lobe↑, cuneus↑ |
| Mayberg e.a. (1992) | Chorea | CMR_glu_ | Grey matter | Depressed patients with early stage HD (n=4) vs. non-depressed patients (n=5) | Orbitofrontal cortex↓, inferior prefrontal cortex↓ |
| Sampedro e.a. (2019) | Chorea | SUV | Pons | Patients with early stage HD (n=18) vs. premanifest mutation carriers (n=21) | Caudate nucleus↓, putamen↓ |
| Young e.a. (1987) | Chorea | CMR_glu_ | Cortex | 1. Patients with stage I (n=9) and stage II (n=8) symptomatic HD vs. persons at risk for HD (n=29); 2. Stage II HD vs. stage I HD | 1. Caudate nucleus↓; 2. Caudate nucleus↓ |
| McDonald e.a. (2006) | Myoclonus | CMR_glu_ | Global mean | Patients with frontal lobe epilepsy (n=18) vs. juvenile myoclonus epilepsy (n=10) | Frontal lobe↓ |
| Schlaug e.a. (1996) | Metabolic disease | CMR_glu_ | Global mean | Patients with Wilson's disease (n=18), who were severely affected (n=?) vs. mildly affected (n=?) | Striatum↓ |
|  |  |  |  |  |  |
| Zhao e.a. (2021) | Auto-immune | SUV | Cerebellum | Patients with LGI1-ab encephalitis with facio-brachial dystonic seizures (n=9), vs. without (n=16) | Putamen↑, left cerebellum↑ |

Results are shown as significant hypermetabolism (↑) or hypometabolism (↓) in the specified region. The following abbreviations are used: SUV: standardized uptake value; CMR_glu_: cerebral metabolic rate of glucose; ROI: region of interest; OPCA: olivopontocerebellar atrophy; MSA(-C): multi system atrophy (of the cerebellar type); UMSARS: unified multiple system atrophy rating scale; SCA: spinocerebellar ataxia; HD: Huntington’s disease; DRD: dopa-responsive dystonia; ET: essential tremor; NBIA: neurodegeneration brain iron accumulation; DBS: deep brain stimulation; (pre)SMA: (pre)supplementary motor area

Supplementary table 4. Overview of articles who performed a correlation analysis with other variables and glucose metabolism.

| Authors (year) | Movement disorder | Patients | Correlation with other clinical variables |
| --- | --- | --- | --- |
| Schöberl e.a. (2017) | Tremor | Patients with orthostatic tremor (n=10) standing | Negative correlation with body sway and metabolism in mesiofrontal cortex. |
| Braun e.a. (1995) | Tics | Patients with Tourette syndrome (n=18) | Positive correlation with complex cognitive and behavioural features and metabolism in orbitofrontal cortex and putamen. |
| Pourfar e.a. (2011) | Tics | Patients with Tourette syndrome (n=12) | Positive correlation with severity of OCD and subject score of OCD pattern (anterior cingulate cortex↓, dorsolateral prefrontal cortex↓, primary motor cortex↑, precuneus↑) |
| Belenky e.a. (2018) | Dystonia | Patients with focal or segmental dystonia (n=10) | Negative correlation with plasma dopamine level and metabolism in temporal cortex. |
| Aguiar e.a. (2016) | Ataxia | Patients with SCA36 (n=20) | No relationship with cognition. |
| Brockmann e.a. (2012) | Ataxia | Patients with SCA17 (n=5) | Positive correlation with cognition and metabolic index |
| Ishibashi e.a. (2017) | Ataxia | Patients with SCA (n=12) subdivided into SCA6 (n=3), SCA19/22 (n=3), sporadic SCA (n=6) | Positive correlation between ^18^FDG uptake en mGluR1 BP_ND_ in cerebellar anterior and posterior lobe and vermis. |
| Meles e.a. (2018) | Ataxia | Patients with SCA3 (n=17) | Positive correlation with letter fluency and SCA-related pattern, not with executive function and information processing speed. |
|  |  |  |  |
| Allain e.a. (2011) | Chorea | Patients with early stage HD (n=8) | Correlations to number of sequencing, boundary, perseverative and intrusion errors of scripts and many cortical areas. |
| Antonini e.a. (1996) | Chorea | Patients with HD (n=8) and asymptomatic gene carriers (n=10) | No correlation with age or CAG number. |
| Berent e.a. (1988) | Chorea | Patients with early to mid-stage HD (n=15) | Positive correlation with verbal learning and memory and metabolism in caudate nucleus; perfomance intelligence quotient with caudate and putamen; no correlation with vocabulary level. |
| Ciarmiello e.a. (2006) | Chorea | Patients with advanced HD (n=47) | No correlation with CAG number. |
| Esmaeilzadeh e.a. (2011) | Chorea | Patients with HD (n=8) | Positive correlation with cognition and metabolism in putamen, caudate nucleus, occipital-, temporal-, parietal- and prefrontal cortex. |
| Kuhl e.a. (1984) | Chorea | Patients with manifest HD (n=13) | No correlation with severity of dementia. |
| Kuwert e.a. (1993) | Chorea | Patients with HD (n=6) | Negative correlation with SEP diagnosis and metabolism in caudate en lentiform nuclei. |
| Martínez-Horta e.a. (2018) | Chorea | Patients with mild HD (n=40) | Negative correlation with apathy and metabolism in the prefrontal cortex, insula, superior temporal gyrus, inferior frontal gyrus, precentral lobe and precuneus. Negative correlation with total functional capacity and superior temporal gyrus, insula and medial frontal cortex; with global cognitive performance and superior temporal gyrus, insula, dorsal anterior cingulate cortex and the medial frontal cortex. |
| Martínez-Horta e.a. (2019) | Chorea | Patients with early-mild stage HD who were bilingual (n=30) | Positive correlation with bilingualism and metabolism in fronto-temporal regions; with Stroop test and dorsal anterior cingulate cortex and left insula; with functional capacity and left inferior orbitofrontal cortex. Negative correlation between irritability and inferior frontal gyrus, superior orbitofrontal cortex and insula. |
| Sampedro e.a. (2019) | Chorea | Patients with early stage HD (n=18) | Negative correlation with apathy and metabolism in left frontal lobe. |
| Squitieri e.a. (2009) | Chorea | Patients with HD treated with placebo (n=12) | Negative correlation with behavioural score and metabolism in frontal and temporal cortex. |
| Young e.a. (1986) | Chorea | Drug-free patients with early to midstage HD (n=15) | Positive correlation with overall functional capacity and caudate and putaminal metabolism. Negative correlation with bradykinesia/rigidity. |
| Berkovic e.a. (1989) | Myoclonus | Patients with myoclonus epilepsy and ragged red fibers (n=5) | No correlation with frequency of tonic-clonic seizures, degree of dementia or duration of illness. |
| Kim e.a. (2005) | Myoclonus | Patients with juvenile myoclonic epilepsy (n=19) | Positive correlation with general spike wave time and metabolism in thalamus, cerebellar hemispheres and midbrain. |
| McDonald e.a. (2006) | Myoclonus | Patients with juvenile myoclonus epilepsy (n=10) | Positive correlation with executive functioning and metabolism in frontal lobe. |
| Kuwert e.a. (1992) | Metabolic disease | Patients with Wilson's disease (n=14) | Negative correlation with degree of pathological reflexes and metabolism in thalamus and cerebellum. |
| Wang e.a. (2021) | Auto-immune | Patients with stiff person syndrome (n=22) or cerebellar ataxia (n=8) associated with anti-GAD65 antibodies | Positive correlation between disability score and metabolism in left inferior lateral posterior temporal cortex. |

The following abbreviations are used: SCA: spinocerebellar ataxia; HD: Huntington’s disease; OCD: obsessive compulsive disorder

Supplementary table 5. Overview of all included articles and methodological aspects.

| Authors | Year of publication | Movement disorder | Participants | Outcome measure* | Minutes after injection | Mean Injection dose MBq | Analysis | Spatial normalization | Intensity normalization | Volume correction |
| --- | --- | --- | --- | --- | --- | --- | --- | --- | --- | --- |
| Aguiar e.a. | 2016 | Ataxia | Patients with SCA36 (n=20) and healthy controls (n=78) | SUV | 45 | 370 | ROI, voxel-wise | Standard SPM template | Global mean | No, but volumetric MRI analysis was performed |
| Brockmann e.a. | 2012 | Ataxia | Patients with SCA17, both manifesting (n=5) and nonmanifesting (n=4), and healthy controls (n=9) | SUV | 40 | 370 | ROI, voxel-wise | Performed but not further specified | Cortical grey matter (excluding region with hypometabolism) | No |
| Gilman e.a. | 1988 | Ataxia | Patients with OPCA (n=30) and healthy controls (n=30) | CMR_glu_ | 30-45 | 185-370 | ROI | Not described | Not described | No |
| Gilman e.a. | 1994 | Ataxia | Patients with sporadic OPCA (n=32), dominantly inherited OPCA (n=19), MSA (n=24), and healthy controls (n=51) | CMR_glu_ | 30-90 | Not described | ROI | Not described | 1: none  2: cerebral cortex | Not described |
| Gilman e.a. | 1995 | Ataxia | Patients with OPCA (n=17), and healthy controls (n=21) | CMR_glu_ | 30 | Not described | ROI | Not described | Cerebral cortex | No, but no strong relationship between atrophy and hypometabolism |
| Grimaldi e.a. | 2019 | Ataxia | Patients with MSA (n=58) subdivided into patients with extrapyramidal, axial, laryngeal-pharyngeal involvement (LPI) and cerebellar symptoms (n = 46)(1), cerebellar and LPI symptoms (n = 30)(2) and cerebellar and cognitive symptoms (n = 9)(3), and healthy controls (n=?) | SUV | Not described | 150 | Voxel-wise | Standard MNI space | Global mean | Not described |
| Ishibashi e.a. | 2017 | Ataxia | Patients with SCA (n=12) subdivided into SCA6 (n=3), SCA19/22 (n=3), sporadic SCA (n=6), healthy controls (n=2) | SUV | 40 | 156 | ROI | Standard SPM template | 1: white matter, 2: global mean | Not described |
| Korinthenberg e.a. | 2004 | Ataxia | Patients with cryptogenic tonic-clonic seizures from infancy (n=30) with ataxia (n=15) | Not described | 30 | 40-60 | ROI and visual | Not described | Global mean | Not described |
| Lee e.a. | 2008 | Ataxia | Patients with multi system atrophy of the cerebellar type (n=41), and healthy controls (n=30) | SUV | 30 | 300 | Voxel-wise | Standard SPM template | Not described | Not described |
| Manes e.a. | 2017 | Ataxia | Patients with SCA38 (n=10) | SUV | Not described | Not described | Voxel-wise | Standardized [^18^F]FDG template for SPM | Iterative global mean | Not described |
| Manes e.a. | 2019 | Ataxia | Patients with SCA38 (n=9) | SUV | Not described | Not described | Voxel-wise | Standardized [^18^F]FDG template for SPM | Iterative global mean | Not described |
| Matthew e.a. | 1993 | Ataxia | Patients with OPCA (n=14) subdivided in affected (n=7) and non-affected (n=7), and healthy controls (n=43) | CMR_glu_ | 30 | 148-185 | ROI | Not described | Grey matter | Not described |
| Meles e.a. | 2018 | Ataxia | Patients with SCA3 (n=17) and healthy controls (n=16) | SUV | 30 | 200 | Network analysis (SSM PCA) | Standard MNI space | Global mean | No, but only weak correlation between grey matter volume and FDG uptake in cerebellar vermis, not in other regions. |
| Mishina e.a. | 1999 | Ataxia | Patients with OPCA in early phase (n=9) and healthy controls (n=10) | SUV | 40 | 150 | ROI | None | Global mean | No, but used ratio which is less effected by PVE |
| Oh e.a. | 2014 | Ataxia | Patients with SCA (n=12) and healthy controls (n=12) | SUV | 40 | 370 | ROI (only cerebellum) | 1: standard MNI space; 2: study-specific cerebellum-template | Not described | Not described |
| Oh e.a. | 2017 | Ataxia | Patients with multi system atrophy of the cerebellar type (n=44), SCA2 (n=9), SCA6 (n=14) and crossed cerebellar diaschisis (n=15), and healthy controls (n=89) | SUV | 40 | 370 | ROI, voxel-wise | Standard SPM template and study specific cerebellar template | Occipital cortex | Yes, Rousset |
| Otsuka e.a. | 1994 | Ataxia | Patients with progressive ataxia (n=11) subdivided into cerebellar cortical degeneration (n=5 (late cerebellar cortical atrophy (n=3), Holmes type hereditary ataxia (n=2)), olivopontocerebellar atrophy (n=6), healthy controls (n=16) | CMR_glu_ | 63-71 | 230-340 | ROI | Not described | Occipital cortex | Not described |
| Rudolf e.a. | 2000 | Ataxia | Patients with inherited or idiopathic cerebellar ataxia (n=23) subdivided into early onset (n=8), late onset (n=9), onset >50 y (n=6), and healthy controls (n=16) | CMR_glu_ | 30-50 | 185 | Visual and ROI | Not described | Global mean | No, as no atrophy was detected |
| Soong e.a. | 2001 | Ataxia | Patients with SCA6 (n=7) and healthy controls (n=7) | CMR_glu_ | Not described | 370 | ROI | Not described | Not described | Not described |
| Tsai e.a. | 2017 | Ataxia | Patients with SCA3 (n=6) and MSA-C (n=1) | SUV | 45 | 370 | Voxel-wise | Standard MNI space | Not described | Not described |
| Volkow e.a. | 2014 | Ataxia | Patients with ataxia-telangiectasia (n=10), non-affected relatives (n=19), and healthy controls (n=29) | CMR_glu_ | 35 | 148-222 | ROI, voxel-wise | Standard MNI space | Both no normalization and normalization to global mean | Not described |
| Wang e.a. | 2007 | Ataxia | Patients with SCA2 (n=8), SCA3 (n=12), SCA6 (n=7), sporadic olivopontocerebellar ataxia (n=3), healthy controls (n=24) | Not described | Not described | 370 | Voxel-wise | Standard MNI space | Global mean | Small volume correction |
| Wang e.a. | 2007 | Ataxia | Patients with SCA2 (n=9), SCA3 (n=12), SCA6 (n=7), healthy controls (n=23) | Not described | Not described | 370 | Voxel-wise | Standard MNI space | Not described | Not described |
| Wüllner e.a. | 2005 | Ataxia | Patients with SCA1 (n=5), SCA2 (n=4), SCA3 (n=6), SCA6 (n=6), Parkinson's disease (n=10), and healthy control (n=10) | Not described | Not described | 400 | ROI, voxel-wise | Standard MNI space | Global mean excluding cerebellum, diencephalon, brainstem and striatum. | Not described |
| Zhao e.a. | 2020 | Ataxia | Patients with MSA-C (n=13), MSA parkinsonism type (n=11) and Parkinson’s disease (n=20), and healthy controls (n=44) | SUV | 40 | 185-370 | Visual, ROI and voxel-wise | Performed but not further specified | Not described | Not described |
|  |  |  |  |  |  |  |  |  |  |  |
| Jang e.a. | 2018 | Autoimmune | Patients with LGI1-ab encephalitis (n=13) of which 69% had faciobrachial seizures (n=9) | SUV | Not described | 5.18/kg | Visual, ROI | Not described | Not described | Not described |
| Kerik-Rotenberg e.a. | 2020 | Autoimmune | Patients with NMDA-ab encephalitis (n=33) of which 58% had a movement disorder (n=19), hyperkinetic (n=6)/mixed(n=17)/hypokinetic(n=3), and healthy controls (n=14) | SUV | 45 | 299 | Visual, voxel-wise | 1: Standard SPM template (PET&VBM module), 2: study specific FDG template | Global mean | Not described |
| Kim e.a. | 2009 | Autoimmune | Patients with Fisher's syndrome (n=10), and healthy controls (n=60) | SUV | Not described | 185 | Voxel-wise | Standard MNI space | Global mean | No |
| Wang e.a. | 2021 | Auto-immune | Patients with stiff person syndrome (n=22, subdivided into classic (n=16), and variant (n=6)) or cerebellar ataxia (n=8) associated with anti-GAD65 antibodies, and healthy controls (n=50) | SUV | Not described | Not described | ROI | Not described | Not described | Not described |
| Zhao e.a. | 2021 | Auto-immune | Patients with LGI1-ab encephalitis (n=25) of which 36% present with facio-brachial dystonic seizures (n=9), and healthy controls (n=44) | SUV | 45-60 | 3.7-5/kg | Voxel-wise | Standard MNI space | Cerebellum | Not described |
| Allain e.a. | 2011 | Chorea | Patients with early stage HD (n=8), and healthy controls (n=12/9) | SUV | 30-50 | 118-280 | Voxel-wise | Normalized space of Talairach | Not described | Not described |
| Antonini e.a. | 1996 | Chorea | Patients with HD (n=8) and asymptomatic gene carriers (n=10) and healthy controls (n=20) | CMR_glu_ | Not described | 112-226 | ROI | Not described | Mean value of plane in which ROI was defined | Not described |
| Bachoud-Lévi e.a. | 2000 | Chorea | Patients with HD (n=5) | CMR_glu_ | 30-50 | 118-281 | ROI | Not described | Global mean | Not described |
| Berent e.a. | 1988 | Chorea | Patients with early to mid-stage HD (n=15) and healthy controls (n=14) | CMR_glu_ | Not described | 185-370 | ROI | Not described | Cortical values of plane in which ROI was defined | Yes |
| Ciarmiello e.a. | 2006 | Chorea | Patients with HD (n=71) subdivided into presymptomatic (n=24) and advanced cases (n=47), and healthy controls (n=30) | SUV | 30 | 300 | ROI | Normalized space of Talairach | Global mean | Yes, Muller-Gartner |
| Esmaeilzadeh e.a. | 2011 | Chorea | Patients with HD (n=8) | CMR_glu_ using an estimated input function | 35 | 200 | ROI, voxel-wise | Normalized space of Talairach | Not described | Not described |
| Feigin e.a. | 2001 | Chorea | Patients with early stage HD (n=13), asymptomatic gene carriers (n=18), and healthy controls (n=8) | CMR_glu_ | 35 | 185-370 | Voxel-wise, SSM/PCA | Normalized space of Talairach | Global mean | Not described |
| Kremer e.a. | 1999 | Chorea | Patients with HD treated with lamotrigine (n=14) or placebo (n=12) | CMR_glu_ | 40 | 111-185 | ROI | Not described | Global mean | Not described |
| Kuhl e.a. | 1984 | Chorea | Patients with manifest (n=13) and at risk for HD (n=15), patients with Parkinson's disease (n=8), healthy controls (n=13) | CMR_glu_ | Not described | Not described | ROI | Not described | Cortical values of plane in which ROI was defined | No, atrophy was not correlated to FDG uptake |
| Kuwert e.a. | 1992 | Chorea | Patients with early HD (n=12), and healthy controls (n=12) | CMR_glu_ | 30 | 185-296 | ROI (only caudate) | Not described | Not described | Yes, Young |
| Kuwert e.a. | 1993 | Chorea | Patients with manifest (n=6) and pre-manifest Huntington's disease (n=12) and healthy controls (n=20) | CMR_glu_ | 30 | 185-296 | ROI | Not described | Cerebellum | Yes, Young |
| Kuwert e.a. | 1993 | Chorea | Patients with manifest (n=20) and pre-manifest HD (n=27) and healthy controls (n=20) | CMR_glu_ | 30 | 185-296 | ROI | Not described | Cerebellum | Not described |
| Lee e.a. | 2012 | Chorea | Patients with manifest HD (n=11) and healthy controls (n=11) | SUV | 30 | 370 | ROI | Standard MNI space | 1: Global mean, 2: white matter, 3: pons | No |
| López-Mora e.a. | 2016 | Chorea | Patients with manifest (n=18) and pre-manifest HD (n=15), and healthy controls (n=18) | SUV | 60 | 277 | Visual, voxel-wise | Standard MNI space | Pons | Yes, using BPM toolbox |
| López-Mora e.a. | 2019 | Chorea | Patients with manifest (n=38) and pre-manifest HD (n=20), and healthy controls (n=18) | SUV | 60 | 277 | voxel-wise | Standard MNI space | 1: Global mean, 2: pons, 3: cerebellum, 4: white matter, 5: thalamus, 6: pons-cerebellar vermis | Not described |
| Martin e.a. | 1992 | Chorea | Patients with HD (n=39), and healthy controls (n=34) | CMR_glu_ | 40 | 111-185 | ROI | Not described | Not described | Not described |
| Martin e.a. | 1995 | Chorea | Patients with early stage HD (n=6), and healthy controls (n=33) | CMR_glu_ | Not described | 110-185 | ROI | Not described | 1: Thalamus, 2: mean value of plane in which ROI was defined | Not described |
| Martínez-Horta e.a. | 2018 | Chorea | Patients with mild HD (n=40) | SUV | 60 | 277 | Voxel-wise | Standard MNI space | Pons-cerebellar vermis | Yes |
| Martínez-Horta e.a. | 2019 | Chorea | Patients with early-mild stage HD who were bilingual (n=30) | SUV | 60 | 277 | Voxel-wise | Standard MNI space | Pons-cerebellar vermis | Yes |
| Mayberg e.a. | 1992 | Chorea | Patients with early stage HD (n=9) subdivided into depressed (n=4) and non-depressed patients (n=5), and healthy controls (n=7) | CMR_glu_ | 35-40 | 185 | ROI | Not described | Grey matter | Not described |
| Mazziotta e.a. | 1987 | Chorea | Asymptomatic patients at risk for HD (n=58), symptomatic patients with HD (n=10), healthy controls (n=27) | CMR_glu_ | 30-40 | 222-370 | ROI | Not described | Cerebral hemispheres including ventricular spaces, and limited to supratentorial regions. | Not described |
| Otsuka e.a. | 1993 | Chorea | Patients with chorea (n=12, subdivided into with dementia (n=7) and without (n=5)): HD (n=5), sporadic chorea with dementia without family history (n=2), chorea-acanthocytosis (n=2), hemichorea caused by vascular lesions (n=2), and healthy controls (n=16) | CMR_glu_ | 63-71 | 200-350 | ROI | Not described | Cerebellum | No, rate of atrophy was associated with hypometabolism, but not in all patients. |
| Paganini e.a. | 2014 | Chorea | Patients with HD (n=10) who received fetal striatal grafting | SUV | 30 | 370 | ROI | Not described | Cerebellum | Not described |
| Sampedro e.a. | 2019 | Chorea | Patients with early stage HD (n=18) and premanifest mutation carriers (n=21) | SUV | 60 | 277 | Voxel-wise | Not described | Pons | Yes, Muller-Gartner |
| Squitieri e.a. | 2009 | Chorea | Patients with HD treated with riluzole (n=11) or placebo (n=12) | SUV | 30 | 300 | ROI | Normalized space of Talairach | Global mean | Yes, Muller-Gartner |
| Young e.a. | 1986 | Chorea | Drug-free patients with early to midstage HD (n=15), and healthy controls (n=14) | CMR_glu_ | Not described | 185-370 | ROI | Not described | Peak cortical activity of plane in which ROI was determined | Not described |
| Young e.a. | 1987 | Chorea | Persons at risk for HD (n=29), patients with stage I (n=9), and patients with stage II (n=8) symptomatic HD, and healthy controls (n=28) | CMR_glu_ | Not described | 185-370 | ROI | Not described | Cortex | Not described |
| Szymanski e.a. | 1996 | Dyskinesia | Schizophrenic patients who developed tardive dyskinesia (n=8), and those who did not (n=8) | CMR_glu_ | 40 | 185 | ROI | Not described | Global mean | No, but volumes were calculated |
| Asanuma e.a. | 2005 | Dystonia | Patients with DRD (n=7), manifesting (n=11) and nonmanifesting (n=12) DYT-TOR1A carriers, manifesting (n=7) and nonmanifesting (n=6) DYT-THAP1 carriers, healthy controls (n=14) | CMR_glu_ | Not described | Not described | Network analysis (voxel based ) | Normalized space of Talairach | Not described | Not described |
| Belenky e.a. | 2018 | Dystonia | Patients with focal or segmental dystonia (n=10) and essential tremor (n=4), and healthy controls (n=53) | SUV | Not described | Not described | Not clearly described, ROI? | Not described | Global mean | Not described |
| Carbon e.a. | 2004 | Dystonia | Patients with a mutation in DYT-TOR1A both nonmanifesting (n=12) as manifesting (n=11), and in DYT-THAP1 both nonmanifesting (n=6) as manifesting (n=7), and healthy controls (n=11) | CMR_glu_ | Not described | Not described | Voxel-wise | Standard MNI space | Not described | Not described |
| Carbon e.a. | 2013 | Dystonia | Patients with a mutation in DYT-SGCE, both manifesting (n=6) and nonmanifesting (n=6), DYT-TOR1A (n=18), DYT-THAP1 (n=13), DRD (n=9), posthypoxic myoclonus (n=7) and healthy controls (n=24) | CMR_glu_ | Not described | Not described | Voxel-wise | Standard MNI space | Cerebral hemispheres | Not described |
| Chase e.a. | 1988 | Dystonia | Patients with idiopathic torsion dystonia (n=6) and healthy controls (n=9) | CMR_glu_ | 30 | 185 | ROI | Each slice scaled to standard with and length | Not described | Not described |
| Eidelberg e.a. | 1995 | Dystonia | Patients with idiopathic torsion dystonia (n=11), and healthy controls (n=11) | CMR_glu_ | 35 | 185-370 | ROI and network analysis (SSM) | Manually to normalized space of Talairach | Global mean | Yes, Rottenberg |
| Eidelberg e.a. | 1998 | Dystonia | Patients with DYT-TOR1A dystonia both manifesting (n=10) and non-manifesting(n=7), and healthy controls (n=14) | CMR_glu_ | 35 | 185-370 | Network analysis (SSM) | Normalized space of Talairach | Not described | Not described |
| Esmaeli-Gutstein e.a. | 1999 | Dystonia | Patients with essential blepharospasm (n=10) and Meige syndrome (n=1) | Not described | 45 | 111-185 | ROI | Not described | Mean value of plane in which ROI was defined | Not described |
| Galardi e.a. | 1996 | Dystonia | Patients with spasmodic torticollis (n=10) and healthy controls (n=15) | CMR_glu_ | 45-70 | 370 | ROI | Manually to normalized space of Talairach | Not described | Not described |
| Gilman e.a. | 1988 | Dystonia | Patients with idiopathic dystonia (n=5) | CMR_glu_ | 30-45 | 185-370 | ROI | Not described | Cerebral cortex | Not described |
| Hutchinson e.a. | 2000 | Dystonia | Patients with essential blepharospasm (n=6), and healthy controls (n=6) | CMR_glu_ | 35 | 185-370 | Voxel-wise | Normalized space of Talairach | Not described | Not described |
| Lalli e.a. | 2012 | Dystonia | Patients with cervical dystonia (n=5) and healthy controls (n=?) | SUV | Not described | Not described | ROI, voxel-wise | Not described | Global mean | Not described |
| Liu e.a. | 2021 | Dystonia | Patients with Meige syndrome (n=50) untreated and right handed, and healthy controls (n=50) | SUV | 60 | 5.55/kg | Voxel-wise and network based (graph theory) | Standard MNI space | Global mean | No |
| Martin e.a. | 1988 | Dystonia | Patients with idiopathic torticollis (n=16), and healthy controls (n=11) | CMR_glu_ | 40 | 185 | ROI | Not described | Not described | Not described |
| Otsuka e.a. | 1992 | Dystonia | Patients with idiopathic dystonia (n=8) subdivided into generalized dystonia (n=3), focal dystonia in the arm (n=3), cervical dystonia (n=2) and healthy controls (n=8) | CMR_glu_ | 63 | 230-460 | ROI | Not described | Not described | Not described |
| Suzuki e.a. | 2007 | Dystonia | Patients with essential blepharospasm (n=25) and normal controls (n=38) | SUV | 45 | 120 | Voxel-wise | Standard MNI space | Global mean | Not described |
| Suzuki e.a. | 2014 | Dystonia | Patients with drug induced blepharospasm (n=21) and essential blepharospasm (n=21), healthy subject using benzodiazepines (n=24), and healthy controls (n=63) | SUV | 45 | 2.5/kg | ROI, voxel-wise | Standard MNI space | Not described | Not described |
| Suzuki e.a. | 2019 | Dystonia | Patients with essential blepharospasm (n=39), healthy controls with closed eyes (n=48) and open eyes (n=48) | SUV | 45 | 2.5/kg | ROI, voxel-wise | Standard MNI space | Global mean | Not described |
| Szyszko e.a. | 2015 | Dystonia | Children with primary dystonia (n=15?), subdivided into DYT-TOR1A (n=2), DYT-SGCE (n=2) and a group without genetic diagnosis (n=12), and neurodegeneration with brain iron accumulation (n=12), | SUV | 30 | 3.6/kg | Visual, voxel-wise | Standard MNI space | Global mean | Not described |
| Berkovic e.a. | 1989 | Myoclonus | Patients with myoclonus epilepsy and ragged red fibers (n=5) and healthy controls (n=6) | CMR_glu_ | 40 | Not described | ROI | Not described | Cerebral cortex in the temporo-occipital area | Not described |
| Frucht e.a. | 2004 | Myoclonus | Patients with myoclonus after cardiac arrest without significant atrophy or focal neurological deficits (n=7), and healthy controls (n=10) | CMR_glu_ using a simplified arterial input function | Not described | Not described | Voxel-wise | Normalized space of Talairach | Global mean | Yes, Meltzer |
| Kim e.a. | 2005 | Myoclonus | Patients with juvenile myoclonic epilepsy (n=19), and healthy controls (n=19) | SUV | 40 | 370 | ROI, voxel-wise | Standard MNI space | Global mean | Not described |
| McDonald e.a. | 2006 | Myoclonus | Patients with juvenile myoclonus epilepsy (n=10), frontal lobe epilepsy (n=18) and healthy controls (n=14) | CMR_glu_ | 35 | 185-259 | ROI | Not described | Global mean | Not described |
| Muccioli e.a. | 2020 | Myoclonus | Patients with Lafora disease (n=8), almost all with myoclonus and ataxia (n=7), and healthy controls (n=?) | SUV | 45-60 | 200 | Visual, voxel-wise | Not described | Not described | Not described |
| Swartz e.a. | 1996 | Myoclonus | Patients with Juvenile myoclonus epilepsy (n=9), healthy controls (n=14) | CMR_glu_ | 30 | 259-370 | ROI | Manually | Global mean | Yes, elimination of portion of regions <2 SD |
| Al-Essa e.a. | 1998 | Storage/metabolic disease | Patients with glutaric aciduria type 1 (n=8) | SUV | 45-60 | 100-370 | Visual | NA | NA | Not described |
| Dubroff e.a. | 2008 | Storage/metabolic disease | Patients with galactosaemia (n=5) and healthy controls (n=8) | SUV | 30 | 5.2/kg | ROI, Voxel-wise | Stereotactic space according to Friston | Not described | Not described |
| Hermann e.a. | 2002 | Storage/metabolic disease | Patients with Wilson's disease (n=37) and healthy controls (n=9) | CMR_glu_ | Not described | 370 | ROI and network analysis (GSOM) | Not described | Not described | Not described |
| Kuwert e.a. | 1992 | Storage/metabolic disease | Patients with Wilson's disease (n=14) and healthy controls (n=23) | CMR_glu_ | 30 | 185-296 | ROI | Normalized space of Talairach | Not described | Not described |
| Lau e.a. | 2021 | Storage/metabolic disease | Patients with Niemann-Pick disease type C (n=14), and healthy controls (n=33) | SUV | 30 | 220-250 | Voxel-wise | Not described | Not described | Not described |
| Schlaug e.a. | 1996 | Storage/metabolic disease | Patients with Wilson's disease (n=18), and healthy controls (n=17) | CMR_glu_ | 45 | 200 | ROI | Not described | Global mean (derived from three planes) | No, however no association with CMR_glu_ and structural abnormalities on MRI |
| Suhonen-Polvi e.a. | 1999 | Storage/metabolic disease | Patients with Salla disease (n=9), four control groups: infants < 1 y with suspected hypoxic-ischemic brain injury (n=12), children 1-2 y and 3-8 y with transient neurologic disorders (n=16), children 9-17 y with migraine without daily medication (n=9), healthy male medical students 24-25 y (n=7) | CMR_glu_ | 0 | 3.7/kg | ROI | Not described | Not described | Not described |
| Braun e.a. | 1993 | Tics | Patients with Tourette syndrome (n=16) and healthy controls (n=16) | CMR_glu_ | 30-45 | 185 | ROI | Not described | Grey matter and average total of ROIs | Yes, Hoffman |
| Braun e.a. | 1995 | Tics | Patients with Tourette syndrome (n=18) | CMR_glu_ | 30-45 | 185 | ROI | Not described | Grey matter | Not described |
| Eidelberg e.a. | 1997 | Tics | Patients with Tourette syndrome (n=10), healthy controls (n=10) | CMR_glu_ | 35 | 185-370 | Voxel-wise, ROI, Network analysis (SSM) | Normalized space of Talairach | Global mean | Yes, Rottenberg |
| Jeffries e.a. | 2002 | Tics | Patients with Tourette syndrome (n=18), healthy controls (n=16) | CMR_glu_ | 30-45 | 185 | ROI | Not described | Global mean | Yes |
| Pourfar e.a. | 2011 | Tics | Patients with Tourette syndrome (n=12) and healthy controls (n=12) | SUV | 35 | 185-370 | Network analysis (SSM PCA) | Performed but not further specified | Not described | Not described |
| Stoetter e.a. | 1992 | Tics | Patients with Tourette syndrome (n=18), healthy controls (n=16) | CMR_glu_ | 30-45 | 185 | ROI | Not described | Global mean | Yes, ROIs placed over local maximum |
| Barath e.a. | 2020 | Tremor | Patients with ET (n=5) before and after treatment with TAPS | SUV | 30 | 296 | Voxel-wise | FDG PET templates from the Mayo Clinic Adult Lifespan Template | Pons | Not described |
| Ha e.a. | 2015 | Tremor | Patients with ET (n=17), and healty controls (n=23) | SUV | 45 | 185-222 | Voxel-wise | Standard MNI space | Not described | Not described |
| Hallett e.a. | 1993 | Tremor | Patients with ET (n=8) and healthy controls (n=10) | CMR_glu_ | 30 | 185 | ROI | Not described | Cerebral hemispheres measured at level of basal ganglia | Not described |
| Reich e.a. | 2016 | Tremor | Patients with ET (n=10) treated with bilateral thalamic DBS of which some developed gait ataxia (n=5) | SUV | 30 | 208 | ROI, voxel-wise | Standard MNI space | Global mean | Not described |
| Schöberl e.a. | 2017 | Tremor | Patients with orthostatic tremor (n=10), and healthy controls (n=10) both standing and lying | SUV | 30 | Not described | Voxel-wise | Standard MNI space | White matter | Not described |
| Song e.a. | 2015 | Tremor | Male patients with ET who responded to medical therapy (n=8) and those who did not (n=9), healthy control male subjects (n=11) | SUV | 45 | 185-222 | Voxel-wise | Not described | Not described | Not described |
| Sun e.a. | 2019 | Tremor | Patients with ET (n=8), parkinsonism (n=38), MSA (n=10), and healthy controls (n=11) | SUV | 60 | 185-370 | ROI | According to Tai et al. 1997 | Cerebellum | Not described |
| Verger e.a. | 2019 | Tremor | Patients with right ET (n=42) before and after left GKVIM (both responders (n=35) and non-responders (n=7)), and healthy controls (n=31) | Not clearly described | 30 | 150 | Voxel-wise | Standard MNI space | Global mean | Not described |

* If no clear statement was made about outcome and no arterial blood sampling was performed, SUV was assumed to be the outcome measure. DBS: deep brain stimulation; ET: essential tremor; GKVIM: Gamma knife of left ventral intermediate nucleus; HD: Huntington’s disease; LGI1-ab encephalitis: leucine rich, glioma inactivated 1, antibody encephalitis; SUV: standardized uptake value; CMR_glu_: cerebral metabolic rate of glucose; MNI: Montreal neurological institute; MSA(-C): multi system atrophy (of the cerebellar type); NMDA-ab encephalitis: N-methyl-D-aspartate receptor antibody encephalitis; OPCA: olivopontocerebellar atrophy; ROI: region of interest; SCA: spinocerebellar ataxia; SPM: statistical parametric mapping; SSM (PCA): scaled subprofile model (principle component analysis); GSOM: Growing Self-Organizing Maps; TAPS: Transcutaneous afferent patterned stimulation.
